# A compendium of synthetic lethal gene pairs defined by extensive combinatorial pan-cancer CRISPR screening

**DOI:** 10.1186/s13059-025-03737-w

**Published:** 2025-09-18

**Authors:** Victoria Harle, Victoria Offord, Birkan Gökbağ, Lazaros Fotopoulos, Thomas Williams, Diana Alexander, Ishan Mehta, Nicola A. Thompson, Rebeca Olvera-León, Stefan Peidli, Vivek Iyer, Emanuel Gonçalves, Narod Kebabci, Barbara De Kegel, Joris van de Haar, Lang Li, Colm J. Ryan, David J. Adams

**Affiliations:** 1 Wellcome Sanger Institute, Wellcome Trust Genome Campus, Hinxton, Cambridge, UK; 2 Department of Biomedical Informatics, The Ohio State University, College of Medicine, Columbus, OH 43210 USA; 3 Centre for Gene Therapy and Regenerative Medicine, King’s College London, 28Th Floor, Tower Wing, Guy’s Hospital, Great Maze Pond, London, UK; 4 European Molecular Biology Laboratory (EMBL), Meyerhofstr. 1 69117, Heidelberg, Germany; 5 Instituto Superior Técnico (IST), Universidade de Lisboa, 1049-001 Lisbon, Portugal; 6 INESC-ID, 1000-029 Lisbon, Portugal; 7 Conway Institute and School of Computer Science, University College Dublin, Dublin, Ireland; 8 Systems Biology Ireland, University College Dublin, Dublin, Ireland; 9 Science Foundation Ireland (SFI) Centre for Research Training in Genomics Data Science, University College Dublin, Dublin, Ireland; 10 The Netherlands Cancer Institute, Plesmanlaan 121, 1066 CX Amsterdam, Netherlands

**Keywords:** CRISPR, Synthetic lethality, Epistasis, High-throughput screening

## Abstract

**Background:**

Synthetic lethal interactions are attractive therapeutic candidates as they enable selective targeting of cancer cells in which somatic alterations have disrupted one member of a synthetic lethal gene pair while leaving normal tissues untouched, thus minimising off-target toxicity. Despite this potential, the number of well-established and validated synthetic lethal gene pairs is modest.

**Results:**

We generate a dual-guide CRISPR/Cas9 Library and analyse 472 predicted synthetic lethal pairs in 27 cancer cell Lines from melanoma, pancreatic and lung cancer Lineages. We report a robust collection of 117 genetic interactions within and across cancer types and explore their candidacy as therapeutic targets. We show that SLC25A28 is an attractive target since its synthetic lethal paralog partner SLC25A37 is homozygously deleted pan-cancer. We generate knockout mice for *Slc25a28* revealing that, except for cataracts in some mice, these animals are normal; suggesting inhibition of SLC25A28 is unlikely to be associated with profound toxicity.

**Conclusions:**

We provide and validate an extensive collection of synthetic lethal interactions across cancer types.

**Supplementary Information:**

The online version contains supplementary material available at 10.1186/s13059-025-03737-w.

## Background

Synthetic lethality (SL) is the concept whereby a cell can survive the loss or mutation of two genes individually, but concurrent loss of both genes results in cell death or a significant loss of cell fitness [1]. In principle, SL interactions can be exploited therapeutically, since somatic alterations that drive cancer development or that are a consequence of the genomic instability that drives tumorigenesis, may create vulnerabilities via the inactivation of a single member of a SL gene pair. In this way tumour cells can be selectively targeted and under optimal conditions the effect of therapy on normal tissues minimised. The success of synthetic lethality as an approach in the clinic is best exemplified by use of PARP inhibitors in the treatment of cancers with deficiency in homologous recombination repair related genes, such as *BRCA1/2* and *RAD51C* [2, 3]. Of note, next-generation PARP inhibitors [4] selectively target PARP1, while having minimal effect on PARP2, avoiding the haematopoietic toxicity associated with PARP2 inhibition while also illustrating that it is possible to selectively inhibit targets, such as single paralogs, even when they have high sequence similarity and related functions.

Large-scale CRISPR-Cas9 screens such as DepMap [5, 6] have identified many essential genes required for the survival of cancer cell lines across diverse lineages. Importantly, although well over a thousand cell lines have been screened with single gRNA (guide RNA) CRISPR libraries, these experiments are under-powered to systematically define all interacting gene pairs. What complicates this analysis further is the complex architecture of the cancer genome and tumour heterogeneity, the limited genetic events captured in available cancer cell lines, the enormous combinatorial space of possible interacting gene pairs, and gene redundancy. Of note, those genes in the genome for which a paralogous gene has been described have been reported to be significantly less likely to be essential, and it is believed that this is due to the phenomenon of “paralog buffering” [7–9].

Several studies have sought to identify novel synthetic lethal gene pairs using dual-guide, also known as combinatorial, CRISPR screening [9–14]. Due to current practical screening limitations, it is not possible to screen all pairs of protein coding genes in parallel, so all such screens have focused on relatively small subsets of protein-coding gene pairs. Paralog pairs have been explored in a number of these studies, leading to the identification of several paralog synthetic lethal interactions, yet despite some overlap between screens most paralog pairs reported as synthetic lethal in these publications appear cell line specific [15–17]. It is important to note, however, that most screens have analysed just two to four cell lines from a diverse range of cancer types/cell lineages, meaning that we lack a comprehensive picture of the pan-cancer synthetic lethal landscape. The aim of our study was to further build upon previous analyses and perform dual-guide CRISPR screens across a large panel of models from different cancer types to identify robust and reproducible SL genetic interactions. Our focus was non-small cell lung cancer (NSCLC), pancreatic cancer, and melanoma; selected as cancers of unmet clinical need (lung and pancreas) and a cancer type that can rapidly progress and for which few effective therapies exist for patients who are unresponsive to or who relapse following immunotherapy (melanoma) [18, 19]. To optimise the relevance of the gene pairs we analysed, we selected candidate synthetic lethal interactions from paralogous gene pairs and gene pairs predict to interact based on integrated analyses of tumours and cell Lines, aiming to enrich for interactions that could be positioned in the clinic. In this endeavour, which is one of the largest of its kind to date, we present data from 472 gene pairs across 27 cell lines and identify a robust set of SL gene interactions, as well as several novel context-dependent hits. We further examine the hits from the screen using multi-parameter imaging and knockout mouse studies.

## Results

### Identification of potential synthetic lethal gene pairs for dual-guide CRISPR screening

**Fig. 1:**
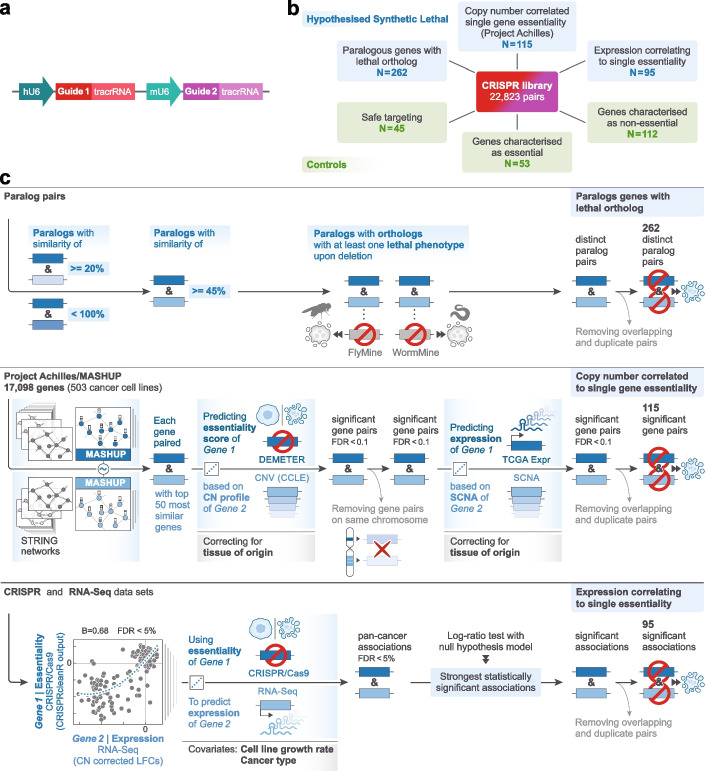
Design and implementation of a dual guide CRISPR library to detect synthetic lethal gene pairs. **A** Schematic of the dual-guide vector used for library construction containing a human U6 (hU6) and a mouse U6 (mU6), two gRNAs and two tracrRNA sequences in a lentiviral backbone (see Methods). **B** The Library contained a total of 22,823 unique gRNAs, targeting 472 gene pairs selected from paralogous genes, MASH-up analysis of Cancer Genome Atlas (TCGA)/Achilles data, and gene pairs derived from an analysis to associate gene expression and CRISPR essentiality (see Methods). Controls included essential/non-essential genes and safe-targeting controls. Each of the abovementioned gRNAs targeting gene pairs were paired with one of the safe-targeting control sequences in the library allowing us to compute the cell fitness effect of single gene disruption. **C** Outlines the methodology used to select the gene pairs analysed in this study. Linear regression associations between CRISPR-Cas9 and RNA-Seq datasets were statistically assessed using log-ratio tests, and multiple hypothesis testing correction was applied using the Benjamini–Hochberg False Discovery Rate (FDR) method

We selected gene pairs for screening from three different sources. Our first selection criteria focused on identifying paralog pair families with two members that had a percentage amino acid sequence identity greater than or equal to 45%. We required these genes to have an ortholog in *Drosophila melanogaster* and *Caenorhabditis elegans* with a lethal phenotype when disrupted in these organisms. We selected genes in this way reasoning that their evolutionary conserved essential function would likely translate into cellular lethality when their orthologs, found as paralogs in human cells, were co-disrupted. We identified these genes and their associated phenotyping data using Flymine [FB2015_15] [20] and Wormbase [WS251] [21, 22] and by using the Ensembl Compara platform [22]. From this analysis we included 262 gene pairs in our library. The second group of gene pairs were identified using a linear regression model between gene-expression and CRISPR-Cas9 essentiality to identify gene pairs that were more likely to be classified as essential in single guide CRISPR screens when a second gene had low/no expression. This analysis involved the use of data from Project Score [23] and was performed across 274 cell lines of diverse origin [11]. Analysis in this way defined 95 candidate gene pairs that were included in the library. Additional paralogous/non-paralogous putative synthetic lethal gene pairs were identified by linear modelling and data integration with MASH-up [24] using pan-cancer TCGA (The Cancer Genome Atlas) [25], expression and copy number data together with data from loss-of-function RNAi-based screens (The Achilles Project) [26], and copy number profiles from the Cancer Cell Line Encyclopedia (CCLE) cancer cell line dataset [27], nominating a further 115 gene pairs. A schematic of the library design is shown in Fig. 1, and the gene pairs are in Additional File 1: Table S1. Further details on the selection of gene pairs are provided in the Methods. To be included in the Library, each of the genes in each gene pair was required to have between 6 and 8 guides, selected to have low off-target scores, a metric of particular importance when targeting closely related sequences such as paralogs [28] (Additional File 1: Table S1). We opted to use a lentiviral dual promoter system consisting of hU6 and mU6 promoters (Fig. 1) engineering this vector to contain two different tracr sequences to reduce the risk of viral recombination. We first tested the positional/promoter effect of the vector by cloning guides (gRNAs) against the cell surface proteins CD15 and CD33 into vectors behind each of the promoters, analysing the results by flow cytometry. We also tested several tracr sequences (Additional file 2: Fig S1) [10, 29]. Although no obvious positional bias was seen at a single guide pair level, to mitigate for this possibility at library scale we placed half of the gRNAs for each gene behind each promoter. In the final vector we also modified the spacer region and tracr sequence to facilitate downstream sequencing and to reduce possible plasmid recombination. The final vector sequence is provided in Additional file 3. In the Library, guides were configured to produce between 18 to 32 guide combinations targeting each gene pair (Additional file 2: Fig S2). In addition to these paired gene/gRNA vectors, each gRNA was paired with a “safe targeting” control selected from Morgens et al., [30]. These gRNAs target regions outside genes that lack any known biological function. Of note, like all guides, these gRNAs generate a double stand break in the genome, but they do not disrupt the function of a gene. This allows us to accurately compute the single gene vs double gene knockout effects and thus to observe interactions between gene pairs. Finally, to calibrate our analysis for defining synthetic lethal relationships, we included guides targeting known essential and non-essential genes [23] within our Library. In total this resulted in an overall Library size of 22,823 unique guide pairs targeting 472 gene pairs, controls and safe-targeting regions (Fig. 1, Additional File 1: Table S1).

### Screening of melanoma, lung and pancreas cell lines

Our Library was used to successfully screen a panel of 27 highly characterised, sequenced and transcriptome profiled, Cas9-positive cell Lines from three cancer backgrounds: 8 melanoma, 10 non-small cell lung cancer (NSCLC) and 9 pancreatic cancer cell lines. Captured within these models were the common driver mutations cognate to these malignancies [5]. All screens were performed in technical triplicate at 1000 × library representation over a 28-day time course and at a multiplicity of infection of 0.3. In addition, we screened three Cas9 wildtype lines (Capan1, A-549 and MeWo), one from each cell Lineage for 7 days to access guide abundance within the library for use as a normalisation control. The growth conditions for each line are provided in Additional File 1: Table S2. After 28 days, cells were pelleted for DNA extraction, and sequencing generated an average of 38 million read pairs per replicate (see Methods). We also assessed Library coverage, which was greater than 300× for most replicates. We next calculated the Gini Index, revealing that most replicates had a Gini score < 0.5 (Additional file 2: Fig S3). The counts matrix is provided in Additional File 1: Table S3.

### Assessing screen performance and identifying synthetic lethal gene pairs

Prior to analysis of our dual guide data, we undertook a series of quality control steps initially assessing the correlation between screens. Samples were excluded if they had a null-normalized mean difference (NNMD) greater than −2. Of note, we had initially screened 29 cell lines, but QC analysis led to the exclusion of two melanoma lines (COLO-792 & WM3702) and four screen replicates, leaving us with 27 lines. Correlation of the control replicates is shown in Additional file 2: Fig S4 and all other QC metrics are shown in Additional file 2: Fig S3. All remaining replicates had BAGEL2 AUC (area under the receiver operating characteristics curve) > 0.88 [31].

**Fig. 2:**
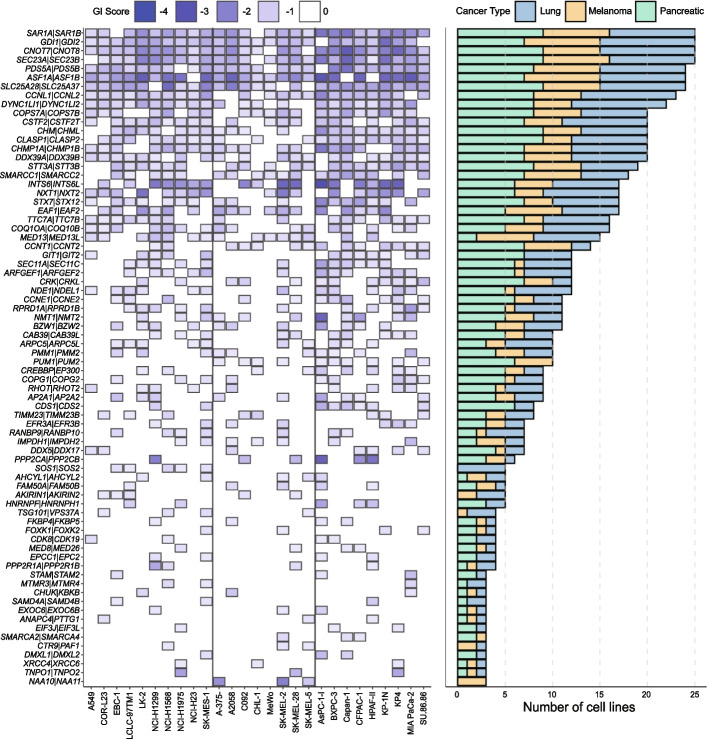
Identification of synthetic lethal gene pairs across 27 cancer cell lines. Heatmap depicting all gene pairs that were classed as synthetic lethal (SL) in 3 or more cell lines. Box colour indicates the mean normalized GI score with pairs considered a hit if the mean normalised GI score was < −0.5 and the Benjamini–Hochberg False Discovery Rate (FDR) < 0.01 (normalised GI scores mean comparison using T-test). Blanks indicate where a gene pair was not a hit in that cell line. Black vertical lines are used to separate cell lines from lung, melanoma, and pancreas. The cell line names are shown on the X-axis. Right: Bar plot shows the frequency that the indicated pairs were a hit with the bar coloured by the cancer type

We next examined the distribution of our essential and non-essential gene controls scaling normalised log_2_-fold changes (LFC) for each cell line by setting the median of the dual safes (control vectors where a safe-targeting control was paired with a gRNA targeting a non-essential gene) to 0 and the median of the single essentials (gRNA targeting a known essential gene paired with a safe-targeting control) to −1. To identify SL gene pairs, we followed a similar analysis method to that employed by Thompson et al. [11] based on the Bliss model [32]. Briefly, we compared the additive effect of each single gene knockout (predicted) to that observed when both genes were targeted simultaneously (observed). The difference between these metrics was determined from Loess regression analysis and termed the residual or genetic interaction (GI) score. A gene pair was considered a SL hit if it had a mean normalised residual (GI) < −0.5 and FDR < 0.01. To remove genes that had a significant effect on growth by themselves, we computed gene essentialities with both MAGeCK [33] and BAGEL2 [31] using the constructs where guides were paired with safe-targeting controls. The number of gene pairs classified as a "hit" per cell line, where the pair is found in three or more lines, is shown in Fig. 2. This analysis shows that there was some variation in the yield of interactions between Lines with the number of hits identified ranging from between 15 (MeWo) to 44 (AsPC-1/SK-MEL-2) (Fig. 2). Additional File 1: Table S4-5.

### Combinatorial screening reveals pan-cancer synthetic lethal gene pairs

Analysis of our screen data as described above identified a robust set of SL gene pairs, with 17 pairs called a ‘hit’ in more than two thirds of tested lines (Fig. 2). Although no gene pair was ubiquitously SL, four pairs were classified as a hit in 25 out of the 27 lines: *CNOT7*|*CNOT8, SAR1A|SAR1B, GDI1|GDI2* and *SEC23A|SEC23B.* Notably, although *CNOT7*|*CNOT8* were not called as a hit in two lines, in both instances this was because they did not pass our stringent hit thresholds for either GI or FDR. In one line the GI was −0.455 (threshold, −0.5) and for the other Line the FDR was 0.066 (threshold < 0.01). Similarly, in two cell lines *SAR1A|SAR1B* and *GDI1|GDI2* were not called a hit because in both cases one of the genes caused single gene lethality, suggesting these genes retain important biological functions not buffered by their paralog partner in these lines (Fig. 2; Additional File 1: Table S4 & S5). Interestingly, few gene pairs were specific to cell lineage, with the notable exception of *GIT1|GIT2* which was a hit in 12 cell lines, but never in a melanoma line. Similarly, *NAA10|NAA11* was only a hit in melanoma lines and never in lung or pancreas cancer models (Fig. 2). This lack of tumour-type specificity is perhaps unsurprising given the selection of gene pairs for inclusion in our library; however, taken together these observations imply that the genes that are a hit in multiple lines have well conserved and critical biological functions regardless of cell background or the driver mutations found in the models we screened.

### Overlap of our data with published combinatorial CRISPR screens

**Fig. 3:**
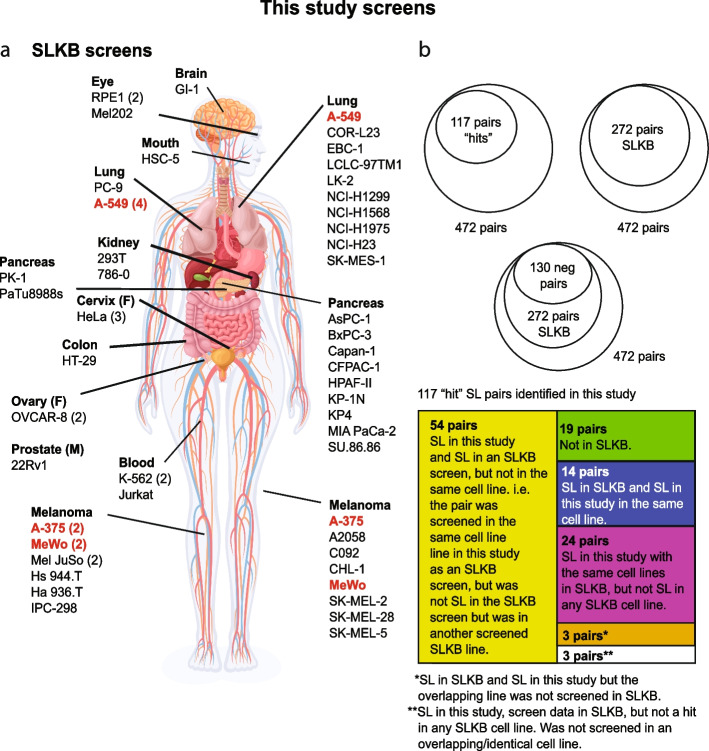
Overlap of our hits and those identified by other published dual guide CRISPR screens. **A** We compared the data from the screens in this paper to those from the Synthetic Lethality Knowledge Database (SLKB) [34]. The screens performed in this study are shown on the right of the body, with those in SLKB shown on the left. Commonly screened cell lines are highlighted in red. The number in brackets next to the cell line name refers to the number of independent screens performed in that line. The anatomical locations are approximate and for reproductive system cancers the sex of the cell lines is shown (M; male. F; female). **B** Shown are diagrams reflecting the overlap of data from the 472 gene pairs screened in this study. We identified 117 screen “hits” across all 27 cell Lines screened. Of these 472 pairs, 272 were found in SLKB and 130 of these pairs were not called as synthetic lethal in our screens or in any SLKB screen (neg pairs). Shown below (box diagram) is a breakdown of the 117 screen hits in comparison SLKB data. The full dataset is available in Additional File 1: Table S6

We next chose to look at the overlap between our data and data from other published combinatorial screens. To do this we used the Synthetic Lethality Knowledge Base (SLKB) [34], which contains data from 22 cell Lines previously screened with combinatorial CRISPR Libraries, including 3 lines also screened as part of this study (Fig. 3). Of the 472 gene pairs we screened, 117 pairs were “hits”, which we defined as synthetic lethal in at least one cell line we screened (Additional File 1: Table S6). In total, we found 882 interactions across the cell Line panel. Of note, 272 of the 472 gene pairs had previously been screened with data deposited in SLKB (we used the author reported data for our analysis). Of these 130/272 were scored as negative (“neg”/no SL interaction) in the screens reported in this paper, which was in complete concordance with data in SLKB. This suggests a very low false negative rate in our study for the detection of epistasis/genetic interactions. Of the 117 hit pairs we identified, 14 were also reported as hits in SLKB for the same cell Line. In contrast, 24 pairs were hits in our study but were not classified as such in another study using the same Line. Finally, we called 54 pairs as a hit pair in a cell Line, and these pairs were also a hit in a screen reported in SLKB, but they were not a hit in the cell Line we screened with data for this cell line and gene pair also available in SLKB. It is notable that some of the abovementioned 54 pairs came from a study previously published by our group [11], with several of these pairs being orthogonally validated by us and in other studies [35]. This discordance likely reflects differences in screen timepoints, analysis methods and hit calling stringency, with other factors such as different culture conditions, gRNA designs, sequencing depth and sequencing/library coverage also likely to be contributing factors. Together with the low pan-screen false negative rate our analysis suggests that our screens have high sensitivity to detect SL interactions across the dataset, but moderate specificity to detect an interaction in a specific cell line. It is notable that a previous comparison of combinatorial screening analysis methods [34] suggested that they exhibit low overlap, and uniformly ascertained datasets such as ours will help to develop better analysis approaches.

### Context dependent SL interactions

**Fig. 4:**
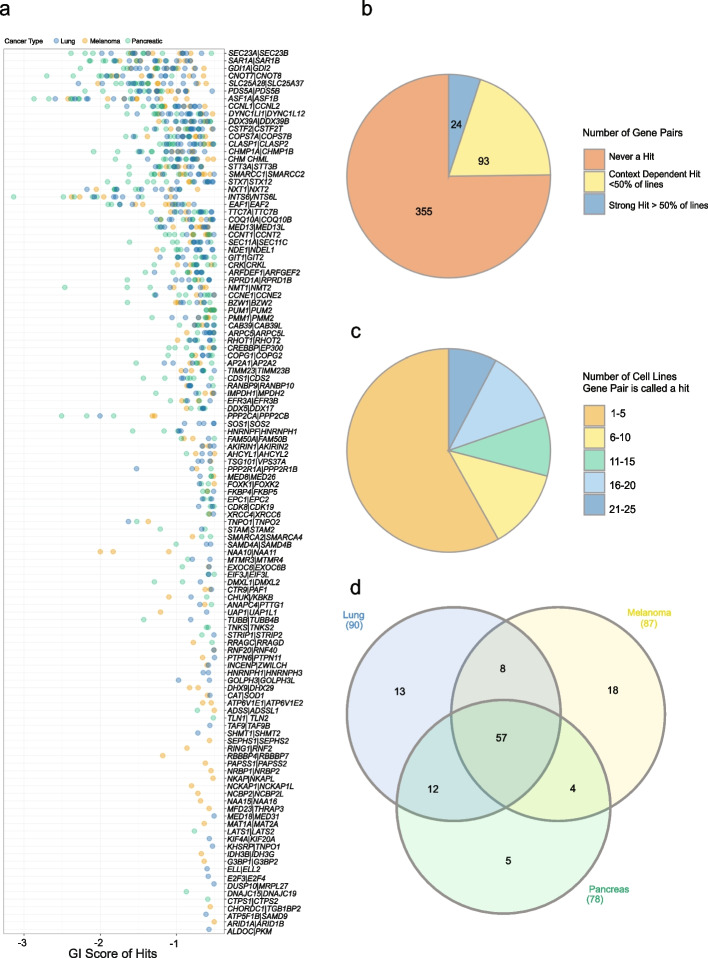
Hits are context specific and not generally associated with cancer type. **A** The mean normalised GI score for each cell line in which the gene pair was classified as a hit is shown. Pairs were considered a hit if the mean normalised GI score was < −0.5 and the Benjamini–Hochberg False Discovery Rate (FDR) < 0.01 (normalised GI scores mean comparison using T-test). Score for each cell line is coloured by cancer type. **B** Pie chart indicating the breakdown of the 472 pairs into never a hit (not a hit in any line), context dependent hit (hit in less than 50% of our cell lines), or strong hit (hit in more than 50% of our cell lines). **C** Pie chart showing the number of lines in which each gene pair was classed as SL (excluding those gene pairs that were not a hit in any line). **D** Overlap of hits by cell line cancer type. This Figure depicts gene pairs that were a hit in one or more cell line

To further explore the overlap of hits between cell lines within our analysis, we plotted the GI score of every hit for every gene pair by tumour type (Fig. 4). Intriguingly, as noted above, despite having data from 472 gene pairs, only 117 were ever a hit. Moreover, only around 5% of our Library was a hit in more than 50% of the cell lines tested (Fig. 2 & 3). Of note, of the 472 gene pairs, at least one gene in 95 pairs was found to be individually essential, and thus the actual search space for genetic interactions was 377 pairs. To further delineate the number of context-dependent gene pairs, we separated the data into groups depending on how often every gene pair was a hit. From this we determined that more than half of our hits occurred in only 1 to 5 cell lines (Fig. 4). Figure 4 shows that this overlap is not determined by cancer cell type. Of note, another factor potentially influencing the results from CRISPR screens is copy number. Currently, there are no well-established methods to correct for this in dual-guide/combinatorial CRISPR screens, so we assessed the possible impact of the copy number landscape on our screen hits, to determine if this could be a variable influencing screen output. In this way we plotted the copy number of our genes, where data was available, revealing variability in ploidy as expected from a panel of cancer cell lines, with the distribution centring around two. This result is comparable to the results of a similar analysis described by Parrish et al., [12]. More specifically, despite a few genes such as *MYC* being heavily amplified (Additional file 2: Fig S5), we note no obvious skew in our results based on copy number, suggesting this is unlikely to be a major contributor to the heterogeneity in SL phenotypes and the GI scores we compute from the analysis of our screens.

### The GI score is an indicator of context independence

**Fig. 5:**
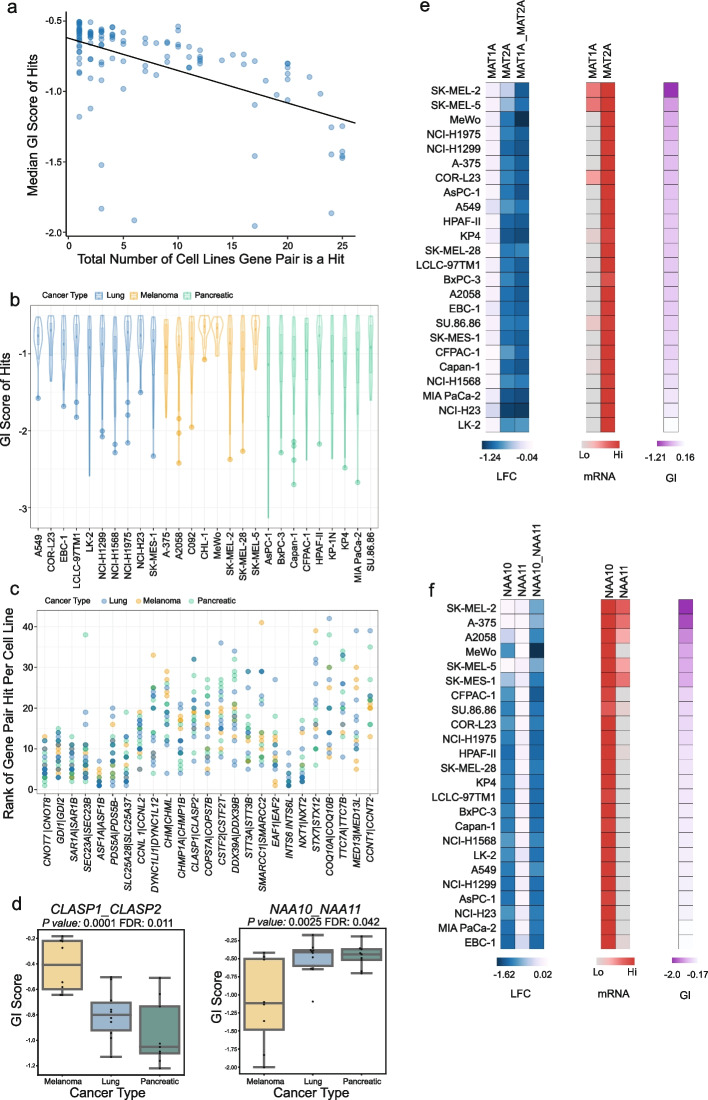
Using GI score and gene pair rankings to define the strongest context independent SL gene pairs. **A** Median GI score per gene pair in which the pair was classed as a hit, plotted against the number of cell lines the gene pair was a hit. Line shows the linear regression analysis with the Pearson’s r correlation coefficient and p-value computed to be −0.557987 and p-value = 6.326e-11, respectively. **B** Range of mean normalised GI scores for hits per cell line. **C** Per cell line each hit was ranked by GI score (lowest GI score for each Line ranked as 1). Graph shows the ranking of each hit per line for the gene pairs classed as strong hits (i.e. a hit in more than 50% of screened lines). **D** The effect of cell lineage on synthetic lethality/GI score. *P* values are derived from a Type II ANOVA analysis with Benjamini–Hochberg correction. **E**–**F** Expression impacts the penetrance of genetic interactions. LFC represents the effect if single gene and double gene disruption. Scaled mRNA levels are shown. GI refers to the GI score

Although we established that most hits from our screen are likely context-dependent, the data in Fig. 4 suggests an association whereby the most common hits have the lowest GI scores. Indeed, every gene pair that had a GI score of < −1.5 in any cell Line was a hit in at least 2 additional cell lines. We hypothesised that the most common SL hits would also be the strongest, and therefore GI scores could be used to predict the likelihood of a gene pair being pan-SL. To test this hypothesis, we plotted the median GI score of the hits for each gene pair against the number of cell lines in which the gene pair was a hit (Fig. 5). The Pearson's correlation coefficient for the median normalised GI versus hit frequency was −0.557987 (*P* < 0.0001), implying a statistically significant negative correlation. We next plotted the distribution of the GI scores of hits by cell line, revealing a dramatic difference between lines with the lowest GI score varying between around −1 and −3 (Fig. 5). This variance between cell lines could explain the low correlation between GI score and hit frequency.

To minimise the impact of cell Line screening efficiency skewing our identification of the strongest hits, we ranked the GI of each gene pair within a cell Line. Taking the gene pairs that we had previously designated as strong hits as they occurred in over 50% of our lines, we next plotted the ranking of each hit (Fig. 5C). This showed a substantial overlap in the top ranked SL gene pairs, with *ASF1A|ASF1B* ranked number 1 in 13 lines, and *INST6|INTS6L* the top ranking hit in 8 cell lines. Most top ranked hits were common across multiple lines. This analysis also highlighted a few interesting context dependent SL gene pairs such as *NXT1/NXT2*, recently described as a synthetic lethal interaction in paediatric cancer models [36].

We noted that much of the variation in genetic interactions across cell lines appeared quantitative rather than qualitative – i.e. rather than a genetic interaction simply being present or absent in specific cell lines, there was variation in the effect size of the genetic interaction across cell lines. To dissect potential causes of this variation, we treated the genetic interaction score as a quantitative trait, similar to how single gene effects have been treated in previous analyses [23].

We tested two possible explanations for variance in genetic interaction score – variation across cancer type (melanoma vs pancreatic vs lung) and variation in the expression of individual genes in each gene pair being targeted. We focussed our analysis on 117 gene pairs that were identified as synthetic lethal in at least 1 cell line. Using a linear modelling approach (see Methods), we found that ~ 20% of these pairs (23 out of 117 at an FDR of 10%; Additional File 1: Table S7) demonstrated variation across cancer types. For example, the paralog pair *CLASP1*/*CLASP2* displayed a much stronger genetic interaction effect in lung and pancreatic models than in melanoma models (*P* = 0.0001, FDR: 0.011; Fig. 5D). Conversely, the pair *NAA10*/*NAA11* displayed a much stronger genetic interaction effect in melanoma models than in lung or pancreatic models (*P* = 0.0025, FDR: 0.042; Fig. 5D). To assess the impact of the expression of individual paralogs on the genetic interaction effect we assessed the correlation between the GI score and the mRNA abundance of each gene in the pair. We found that, at an FDR of 10%, variation in the GI score of 7 gene pairs could be associated with variation in the mRNA abundance of at least one gene in the pair. For instance, the genetic interaction between *MAT1A/MAT2A* was only evident in cell lines where *MAT1A* was expressed at higher levels (Pearsons = −0.6657, *P* = 0.045; Additional File 1: Table S7). In cell lines with no, or extremely low, expression of *MAT1A*, then *MAT2A* was individually essential, and no genetic interaction was observed (Fig. 5E).

We note that cancer type specific effects and variation in gene expression are not mutually exclusive explanations for variation in genetic interaction effects – some gene pairs are likely synthetic lethal in specific cancer types due to cancer-type specific variation in the expression of members of the gene pair. For instance, *NAA11* displays low expression in pancreatic and lung cell lines and, likely as a result, its paralog *NAA10* appears to be individually essential in these models. In contrast, in melanoma models *NAA11* is highly expressed and *NAA10* is not individually essential, but in these models the combined disruption of *NAA10/NAA11* causes a significant growth defect (Fig. 5F).

Previous work has established that there can be significant variation in the synthetic lethality of paralogs across cancer cell lines [11, 15, 16]. Our results here suggest that variation in mRNA expression of the paralogs themselves is one contributing factor.

### Phenotypic validation of pan-cancer synthetic lethal gene pairs

**Fig. 6:**
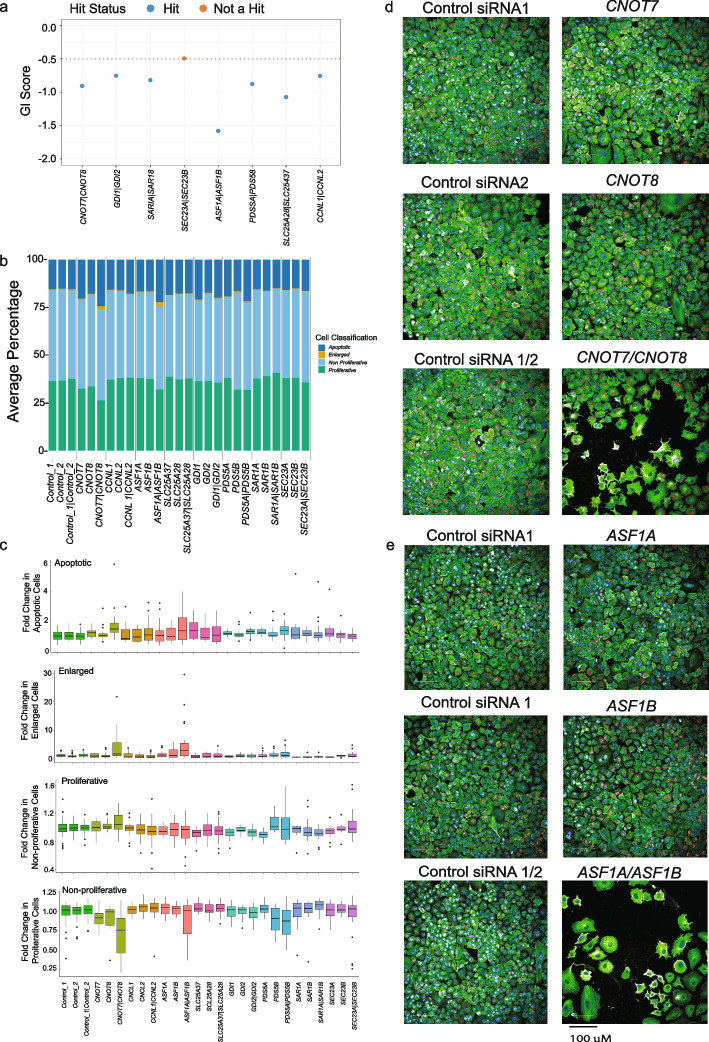
Phenotypic validation of strongest hits by siRNA knockdown and subsequent cell imaging. **A** GI scores in the A549 cell line for the pairs analyses by imaging. **B** Percentage of cells defined as apoptotic, enlarged, non-proliferative and proliferative. **C** Fold change in apoptotic, enlarged, non-proliferative and proliferative for genes and gene pairs analysed in this experiment. The data represent the median of four independent experiments with the lines and bars the IQRs. **D** Representative pictures of cellular phenotypes observed following gene co-disruption. Colours are as follows: DNA (blue), tubulin/spindles (green), Annexin V/apoptosis (red)

To further explore the gene pairs we identified, we took our top gene pairs, those that were a hit in 23 or more cells lines, and performed phenotypic analysis on cells in which we had supressed their expression using siRNA. More specifically, our aim was to determine the phenotypic effects of these pairs and determine if they reduced cell growth and/or increased cell death using orthogonal techniques and to explore the underlying biology/mechanisms of synthetic lethality. To achieve this, we knocked down the gene pairs using siRNAs in A549 cells, selecting this model because of its ease of imaging and because the interactions to be tested were found in this line by CRISPR screening (Fig. 6A). Analysis was performed 72 h after siRNA transfection where cells were stained with markers of proliferation (EdU) and apoptosis (Annexin V), as well as markers of cell morphology (Hoechst, F-actin and α-tubulin) with cells imaged using the Operetta system. Analysis was performed using a machine learning algorithm implemented to categorize cells as proliferative, non-proliferative or apoptotic, or as having other phenotypes. Of note, our approach aimed to capture the acute effects of gene silencing, where scorable, but since the original CRISPR screens were performed over several weeks, late effects beyond the 72 h timepoint will not be visible. Importantly, during pilot experiments we observed several conditions that led to the appearance of atypically “large cells”, so we added this as an additional classification category to our screen analysis. In Fig. 6B we show the average percentage of cells per siRNA treatment that were classified into each of the abovementioned categories. These data are displayed as percentages and show cellular phenotypes including decreased proliferation and increased numbers of enlarged cells for several gene pairs including *CNOT7/CNOT8, ASF1A/ASF1B* (Fig. 6C-E). Of note, several significant gene pairs from the CRISPR screens had no obvious effect on cell growth or other phenotypes. This Likely reflects the proximal timepoint of analysis at 72 h after siRNA transfection, with the cell fitness phenotypes we observed in our CRISPR screens occurring after 28 days in culture. This observation may explain why previous attempts to identify synthetic lethal interactions using siRNA/shRNAs have been problematic and lower return when compared to CRISPR-based approaches [37].

### Positioning of SL gene pairs

**Fig. 7:**
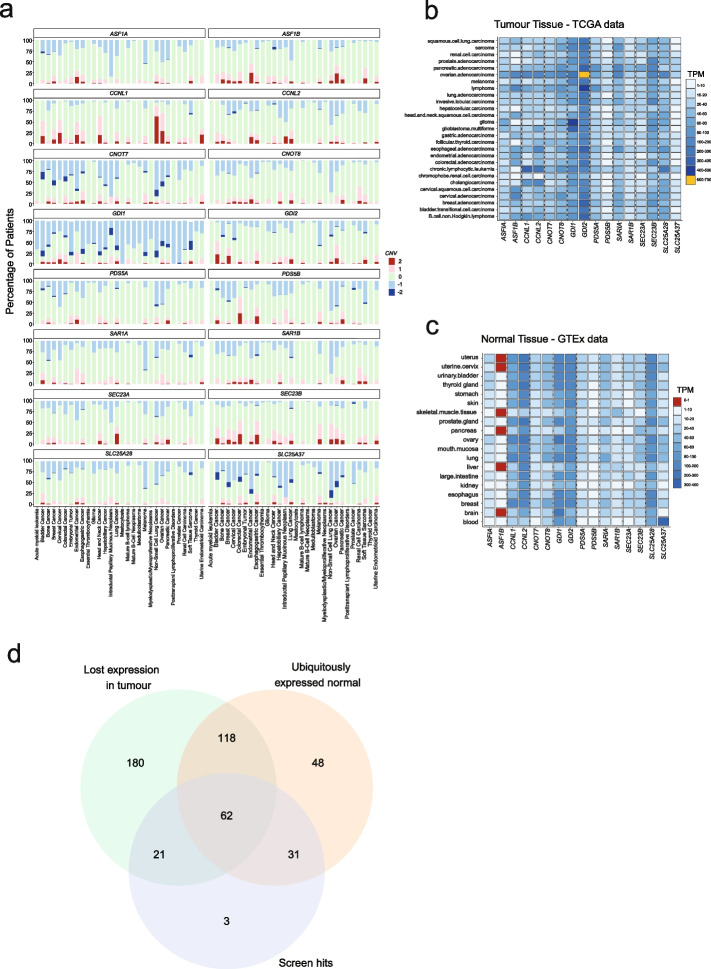
Positioning of synthetic lethal interactions via comparison to human cancer and normal tissue datasets. **A** Analysis of pan-cancer copy number profiles for selected synthetic lethal gene pairs with the data expressed as a percentage frequency for each TCGA cancer type. The data was downloaded from the Xena Browser [45] and has been described previously [46]. Dark blue denotes homozygous loss of each gene by cancer type. **B** Expression of synthetic lethal genes across cancer types. Shown are the transcripts per million values. **C** Expression of synthetic lethal genes across normal tissues. Shown are the transcripts per million values. The data was downloaded from GTEx [38]. **D** We identified 62 gene pairs for which we observed ubiquitous expression in normal tissues, loss of expression of one member of the pair in tumours (left), where the pair was also found to be a screen hit. Expression was defined as > 1 TPM

While the primary goal of our study was to identify epistatic synthetic lethal interactions at scale, translation of these results such that candidates may be targeted therapeutically is of great interest. To advance this we used pan-cancer genomic data derived from 30 studies to explore the landscape of gene disruption by copy number alteration, and the expression of gene pair candidates using data from both GTEx [38], representing normal tissues, and the Tumor Genome Atlas (TCGA) [25]. As shown in Fig. 7, for several genes, most notably *CNOT7, GDI1* and *SLC25A37*, there are a subset of tumours that showed homozygous deletion and thus inhibition/suppression of *CNOT8, GDI2* or *SLC25A28*, respectively, would be predicted to provoke cellular lethality/reduced cancer cell fitness. Similarly, cases with homozygous deletion of one member of a SL gene pair were found for the other candidate pairs, albeit at a lower frequency. Since our screening efforts had focused on tumour cell lines, we were mindful that one factor that might preclude the candidacy of a SL interaction for therapeutic development is whether loss/silencing of the target is observed in normal cells/tissues. Although some genes such as *PDS5B* were expressed at low levels across normal tissues, *ASF1B* was the only gene whose expression was convincingly absent. This included brain, liver and pancreas with this expression profile suggesting that *ASF1A* inhibitors should be developed with caution. Extending this analysis further we identified 62 gene pairs for which we observed ubiquitous expression in normal tissues, loss of expression of one member of the pair in tumours, where the pair was also found to be a screen hit (Fig. 7). Further triaging of these candidates may reveal paradigms for further drug development. Given our abovementioned desire to minimise the risk that therapeutic candidates were toxic to normal cells we next asked if any of these 62 genes had been disrupted as part of the Sanger Mouse Genetics Project, where null alleles in mice had been extensively phenotyped for > 200 traits [39]. In this way we found knockouts for several genes including *Slc25a28* and *Cnot7*. Knockouts of further genes were extensively phenotyped across the International Mouse Phenotyping Consortium [40] (Additional File 1: Table S8). We were drawn to focus on a knockout of *Slc25a28*, noting that this mutant line had been scored as having cataracts in some mice, but no other phenotypic traits (Additional file 2: Fig S6). In keeping with this, *SLC25A28* has a pLI (predicted probability of loss-of-function) score of 0.85 in Gnomad [41] suggesting moderate tolerance of transcript/gene loss; essential genes have a pLI of ~ 1. Finally, we next aimed to determine which of the pairs we screened had compounds already designed against them, including those used in clinical trials, or if they potentially represent tractable targets for therapy. To do this we used the COSMIC actionability classifications [42] and the Pharos target designations for each gene/target. Pharos classifications [43] include four ranks Tclin, Tchem, Tbio and Tdark. These ranks denote targets that are clinically established targets, those that have chemical modulators available that have already been developed, proteins with well-established biological roles but no known chemical modulators or approved drugs, or understudied or unknown targets, respectively (Additional File 1: Table S9). For some of the pairs in our library chemical compounds have already been developed. For *SLC25A28* specifically, no compounds have been synthesized yet, but SLC25A28 has been reported to be in the mitochondrial inner membrane where other agents have been developed [44].

## Discussion

PARP inhibitors are a well-tolerated and effective anti-cancer therapy that rely on defects in genes controlling homologous recombination to create a cellular vulnerability. In recent times, clinical trial data has suggested these agents could be used for prophylaxis in germline carriers of pathogenic *BRCA1/2* alleles [47]. The development of additional synthetic lethal strategies for cancer management is therefore of great interest.

In this paper, we describe one of the largest combinatorial CRISPR studies performed to date, defining 117 synthetic lethal gene pairs which we further position by comparison to human cancer genome datasets; by functional experiments in vitro, and by exploring the phenotypes of knockout mice. There are several notable aspects of our study. Firstly, using the strategy of selecting gene pairs described here we observed that few of the hits were unique to any one cell lineage/tumour type. Since each of the tumour types we screened have a different cell of origin, different driver mutations and genomic structures, it seems likely that in most cases interactions are not readily predictable from the genome sequence or tumour type alone, at least for the candidate SL pairs we screened. This is in keeping with analyses of DepMap where a limit number of vulnerabilities of large biological effect have been conclusively associated with tumour type or genomic features, the exception being the interaction between mismatch repair deficiency and *WRN* [48], and the targeting of tumour type or lineage-specific oncogenes. This is at least in part an issue of power, where not all cancer-associated genomic features are captured in available cell line models, but it also reflects the complexity of defining these interactions. The gene pairs we examined here were ascertained from multiple analyses yet paralog pairs gave the highest yield of SL interaction, as we have described previously [11]. Reassuringly, as part of this study we identified several gene pairs such as *SLC25A37/SLC25A28* and *CNOT7/CNOT8* that represent attractive targets for further drug development, with *Slc25a28* knockout mice being viable and fertile with no overt phenotypes, other than cataracts in some mice.

## Conclusions

Our dataset will facilitate benchmarking activities which will be important for the development of better screen analysis algorithms and alongside the large collection of synthetic lethal pairs, will provide new interactions with which to explore the biology of epistasis.

## Methods

All code and notebooks used in this study are available in Github [67] and Zenodo [49]. All code is covered by an MIT license.

### Public data retrieval

For 503 cancer cell lines included in the Cancer Cell Line Encyclopaedia, we downloaded publicly available shRNA screening-based gene essentiality data (represented as DEMETER-based Z-scores: ExpandedGeneZSolsCleaned.csv) from the Project Achilles Data Portal (http://portals.broadinstitute.org/achilles/, v2.20.2) [50]. For these same cell lines, we also downloaded publicly available SNP array-based gene-level copy number profiles (CCLE_copynumber_byGene_2013-12–03.txt) from the Cancer Cell Line Encyclopedia Data Portal (now accessible as CCLE legacy data at https://data.broadinstitute.org/ccle_legacy_data/dna_copy_number/) [27]. Gene-specific vectors that integrate the human STRING network (v9.1) using the Mashup algorithm were obtained from the supplementary data of the original publication (string_human_mashup_vectors_d800_V1.txt) [24]. Additionally, SNP array-based copy number profiles (computed using the GISTIC algorithm [51]) and RNA-seq based gene expression data for 7,537 patients/tumours from The Cancer Genome Atlas (TCGA; see Additional File 1: Table S10 for patient IDs) were accessed through the Broad Firehose Analysis Pipeline in March 2018. Further datasets are described in each of the methods sections below.

### Identification of synthetic lethal gene pairs

Predicted SL gene pairs were identified from three sources (Fig. 1). Firstly, to identify potential SL gene paralogs 19,781 protein coding genes from Ensembl v92 were scanned for paralogs with > = 45 percent reciprocal sequence identity. Flymine [52] and WormBase [21] were used to identify corresponding orthologs with a lethal phenotype upon deletion/depletion in *Drosophila melanogaster* and *Caenorhabditis elegans* (annotated Paralog)*.* Secondly, we used a multi-step statistical approach to predict SL genes by: 1). For every gene, we identified the top 50 functionally most similar genes, by calculating gene–gene Pearson correlations among Mashup-based gene-specific vectors representing an integration of the human STRING networks [24]. As shown previously [24] this strategy identifies gene pairs with highly similar biological functions (including but not limited to paralogs), which are strongly enriched for genetic interactions. 2). For the resulting potentially interacting gene pairs, we next employed ordinary least squares regression (using the Python package Statsmodels (https://www.statsmodels.org), on the Achilles dataset [26] of 503 cancer cell lines. This was to assess if copy number loss (defined as a copy number score < −0.3, GISTIC’s default noise threshold for deletions) of one gene in a pair correlates with increased essentiality (indicated by a lower DEMETER-based Z-score) of the partner gene, while adjusting for the tissue of origin. Gene pairs located on the same chromosome were excluded because they share similar copy number dynamics, which could falsely suggest significant interactions due to increased sensitivity to shRNA knockdown when the gene is (heterozygously) deleted. From this analysis, we identified 357 significant gene pairs (Benjamini–Hochberg FDR < 0.1). 3). Finally, for these 357 pairs, we used ordinary least squares regression to investigate, in a pan-cancer context using data from 7,537 patients/tumours in TCGA, if the deletion of one gene (again defined as a GISTIC copy number score < −0.3) was associated with a significant upregulation of RNA expression of the partner gene. This analysis yielded 125 significant (Benjamini–Hochberg FDR < 0.1) computationally derived putative synthetic lethal gene pairs (115 were included in the library). Thirdly, a linear regression model was used to identify associations between corrected LFCs from CRISPR/Cas9 single knockout screen data and RNA expression data. Cell line growth rate and cancer types were included as covariates. A log-ratio test with a null hypothesis model was used to determine statistically significant associations and the strongest of these taken forward and included in the library. For controls, we included guides targeting established non-essential and essential genes [23], as well as several safe targeting guides [30].

### gRNA selection and library design

We selected 8 guides per gene; 4 from the Brunello library [53] and 4 from the Toronto library [54]. When 4 guides from these sources were unavailable a guide was selected from the Lander-Sabatini library [55] or designed in house using the Broad GPP server (https://portals.broadinstitute.org/gpp/public/). Where a minimum of six guides were not available, the gene was excluded from the library. Additional File 1: Table S1 contains on-target/off-target scores for all gRNAs, these calculations are detailed below. A variant of this library has been published previously [56].

### Library cloning

Library cloning was undertaken following the general protocol of Vidigal and Ventura [57] with some modifications to the vector to replace the second sgRNA scaffold and modify the region between the first scaffold and second promoter to prevent spurious primer binding. Oligo sequences were ordered from TWIST Bioscience with the following sequence and designed to modify the second scaffold to a sequence taken from Shen et al., [10]. We resuspended the synthesised TWIST oligo pool to a concentration of 0.01 ng/µl and amplified it using Q5 High-Fidelity 2X Master Mix (NEB, #M0492). 24 × 50 µl PCR reactions were performed with conditions as follows: initial denaturation of 98 °C for 30 s, 17 cycles of 98 °C for 10 s, 68 °C for 35 s and 72 °C for 30 s and a final 72 °C extension for 10 min. The resulting products were purified using a PCR Cleanup Kit (Monarch T1030S).

We obtained a modified pDonor_mU6 vector from GeneArt and digested it with BbsI (NEB R3539S). Linearised vector was excised from an agarose gel, purified using a DNA gel extraction kit (Monarch T1020S) and the oligo library cloned using NEBuilder® HiFi DNA Assembly Master Mix (NEB E2621S). The Gibson product was then digested using BbsI and gel purified. It is important to note that because this Ligation and Linerisation strategy results in complementary stick ends, it is possible for guides to form concatemers prior to BbsI digest. As most guide pairs in our library are unique, BbsI cleavage will result in guide swapping and a subsequently high guide mismatch rate. We were aware of this problem from previous libraries and thus we increased our screen representation to 1000× to compensate for the reads that are discarded due to mismatching.

The lenti-guide-puro backbone plasmid (Addgene #52,963) was digested using BsmBI (NEB R0580S), dephosphorylated using Antarctic Phosphatase (NEB M0289S) and the linerised vector was excised from a gel. The cloned oligo fragments now containing two guides separated by the first guide scaffold and mU6 promoter were cloned into the linerised lenti-guide-puro vector using the quick ligation kit (NEB M2200). The Library was purified and electroporated into NEB 10-beta electrocompetent *E.Coli* (NEB C3020); 10 electroporation reactions were performed each with 25 µl of bacteria and 2.5 µl library. Serial dilutions were plated on ampicillin agar plates to assess library representation and the remainder of the library expanded in liquid culture containing ampicillin overnight. Bacteria was pelleted and plasmid DNA extracted using the Giga prep kit (Qiagen 12,991). The sequence of the final vector is provided in Extended Note 1.

### Cell culture, lentivirus production and cell screening

All cell lines were obtained from ATCC except for CO92, which was from Dr. Nick Hayward (QIMR, Brisbane). All cell lines were STR profiled to confirm authenticity. None of the cell lines used have previously been misidentified [58]. Cell Lines were cultured in DMEM or RPMI with 10% FBS except for HEK293T, which were cultured in IMDM (Additional File 1: Table S2). L-Glut was added as required. All Lines were confirmed to be mycoplasma negative and cultured at 37 °C in 5% CO_2_. To make lentivirus 1.8 × 10^7^ HEK293T cells were transfected with 4 μg pMD2.G (Addgene #12,259), 18.5 μg psPAX2 (Addgene #12,260) and 7.5 ug/ml transfer vector. Media was Changed within 16 h of transfection, virus was filtered with 0.45 μm low protein-binding filters after 30 h, aliquoted, and stored at −80 °C.

Cell lines were made Cas9 positive by infecting with lentivirus produced with pKLV2EF1a-Cas9Bsd-W plasmid (Addgene #68,343) and selected with blasticidin (InvivoGen). Cas9 activity was assessed to be over 90% using a BFP/GFP reporter assay using pKLV2-U6gRNA5(gGFP)-PGKBFP2AGFP-W (Addgene #67,980) and pKLV2-U6gRNA5(Empty)-PGKBFP2AGFP-W (Addgene #67,979), as described by Tzelepis et al., [59].

For screening, the library virus was titrated using puromycin selection and an MTS assay (Promega G3580) to assess cell viability. Then, cells were infected in suspension using virus at an MOI of 0.3 and Polybrene. Media was Changed the following day and puromycin added 24 h later. Infections were conducted at 1000 × representation and cells maintained at 3000 × representation for the duration of the screen. Cells containing Cas9 were harvested 28 days post infection, control Cas9 null cells were harvested at 7 days.

### DNA extraction, PCR and sequencing

DNA was extracted from cell pellets using the Gentra Puregene Cell Kit (Qiagen 158,388). 50 × 50 µl PCR reactions were performed per sample/replicate each containing: 3 ug genomic DNA, 25 µl 2 × KAPA HiFi HotStart ReadyMix (Roche KK2602) and primers listed in Additional File 1: Table S11. Products were size selected using SPRIselect (Beckman Coulter B23317) at a ratio of 0.6. Four second round PCR reactions were performed per sample, each reaction contained: 200 pg PCR product, 25 µl KAPA and Illumina tags (Additional File 1: Table S11). PCR was run with a maximum of 10 cycles, reactions were combined and 46 µl taken for SPRIselect purification again at a ratio of 0.6. The purity and concentration of products was determined using a Bioanalyser (Agilent). Paired end sequencing was performed using Illumina HiSeq 2500 with the custom primers listed in Additional File 1: Table S11.

### Screen data analysis

#### Analysis of CRISPR Screening data

Quantification and pre-processing.

Unaligned CRAMs were download from the ENA (project ERP145919) and quantified using pyCROQUET version 1.5.1 (https://github.com/cancerit/pycroquet) with the dual-guide subfunction, with -chunks 50,000 -b exact as non-default options. Paired guide counts were summarised by sample and collated into a count matrix. Sixty user-defined guide pairs with high read counts were removed prior to total normalisation by adding a pseudocount of five, dividing by the total reads mapped per sample and a scaling factor of 10 million. Fifty-one guides were removed with less than 20 reads per 10 million in all control samples before calculating the normalised log2 fold changes.

#### Quality control and scaling

Spearman’s rank correlation coefficient was used to assess the correlation between replicates while the null-normalised mean difference (NNMD) was used to measure the separation between Essential and Non-essential single guide pairs.

Where:

*μE* is the mean of the single essential guides.

*μNE* is the mean of the single non-essential guide pairs.

*σNE* is the standard deviation of the single non-essential guide pairs.

Samples with a NNMD > −2 or R < 0.55 were excluded from further analysis: KP-1N R1 (Pancreas), COR-L23 R2 (Lung NSCLC), NCI-H1568 (Lung NSCLC), SK-MEL-5 R1 (Melanoma), SK-MEL-28 R2 were excluded in addition to all three replicates from WM3702 (Melanoma) and COLO 792 (Melanoma).

Normalised log2 fold changes were scaled such that the median of the double safe-targeting guide pairs was zero and the median of the single essential guide pairs was −1.

#### Single gRNA analysis

Dual guide pairs, where both guides were gene-targeting (gene|gene) were excluded for the single guide analyses such that only single and control guide pairs were carried forward (gene|safe_targeting or safe_targeting|gene and safe_targeting|safe_targeting, respectively). Scaled counts were compared to the mean of the controls to identify enriched and depleted genes using MAGeCK version 0.5.9.3 with the options –norm-method'none’ and –remove-zero'none’. Genes were classified as enriched with a positive FDR < 0.05 and depleted with a negative FDR < 0.05. Scaled fold-changes were used to identify depleted genes using BAGEL2 version 2.0 build 114. Core essential (CEGv2) and non-essential (NEGv1) gene lists for BAGEL2 were sourced from the Hart Lab repository (https://github.com/hart-lab/bagel, build 114), containing 684 and 927 genes respectively. For each dataset and cell line, scaled Bayes factors were calculated by subtracting the threshold calculated with the R pROC package such that genes considered as depleted had a scaled Bayes factor > 0. For each gene pair, the gene in position A (targetA) or position B (targetB) was classified as being ‘single_depleted’ if the gene in the relevant position had both a MAGeCK neg.fdr < 0.05 and a BAGEL2 scaled BF > 0.

#### Dual gRNA analysis

Within the library, a gene pair is comprised of guide pairs testing the interaction between two genes (geneA|geneB and geneB|geneA). These guide pairs are classified as:

- singles—where one guide is gene-targeting and the other is safe-targeting (gene|safe_targeting or safe_targeting|gene).

- duals—where both guides are gene-targeting (gene|gene).

- safe-targeting—where both guides are safe-targeting (safe_targeting|safe_targeting).

Within each sample, the observed fold changes were normalised by subtracting the median fold change of the singles. The Bliss independence model was used to calculate the expected combined effect of two genes being targeted simultaneously as follows:

Dual expected FC (*g1g2*) = Single observed FC (*g1*)—Single observed FC(*g2*).

where *g1g2* represents the dual construct which contains two gene-targeting guides and *g1* and *g2* correspond to single constructs containing the same gene-targeting guides paired with a safe-targeting guide.

Using Loess regression, we modelled the relationship between predicted and observed fold changes to determine a gene interaction (GI) score (or residual) for each dual guide pair. As observed in Thompson et al., [11], variance was not equal across the model showing increased variance in gene pairs exhibiting greater lethal effects. To account for this heteroscedasticity, we applied variance smoothing. Dual guide pairs were ranked by their expected fold Changes and their residuals were placed into batches of 200 guide pairs. A normalised GI score (norm_gi) was then calculated by dividing each residual by the square root of its corresponding batch variance. For each gene pair, a t-test was performed using the pooled variance of the normalised GI scores, comparing the median normalised GI score of each gene pair to the overall median and applying Benjamini–Hochberg to control false discovery rates.

For each cell line, gene pairs with a mean normalised GI < −0.5 and FDR < 0.01 and significant by the Bassik method were taken forward. Furthermore, pairs were only classified as hits if neither gene was categorised as'essential'by both BAGEL2 and MAGeCK (i.e. significantly depleted in both single-gene analyses).

#### Cell imaging

A549 cells were reverse transfected with a total of 50 nM siRNA using 0.2 µl DharmaFECT 1 transfection reagent (Horizon Discovery T-2001–02) and seeded onto glass bottom plates (Grenier, 655,891). For each gene target four individual siRNA sequences were used, one per well. For dual knockdowns 25 nM siRNA against each target was combined, for single knockdown 25 nM targeting siRNA was combined with 25 nM safe-targeting control 1 siRNA.

After 72 h cells were treated with 10 µM EdU (Thermo Fisher C10639) and incubated at 37 °C for 2.5 h prior to treatment with 1 µg/ml Hoechst 33,342 and incubation for an addition 30 min. Cells were washed well with PBS and treated with Annexin V conjugate 647 (Thermo Fisher A23204) at 1:10 dilution in 1 × Annexin binding buffer (BD Bioscience 556,454). After 15 min incubation cells were PBS washed and fixed with 4% paraformaldehyde for 15 min prior to permeabilization with 0.5% Triton® X-100 in PBS for 20 min. Cells were washed with 3% BSA and EdU detected by the addition of 50 µl of the Click-iT® Plus reaction cocktail (Thermo Fisher C10639) and incubation for 30 min. Cells were blocked with 1% Goat serum and 3% BSA in PBS for 1 h and then incubated overnight at 4 °C with alpha tubulin antibody (Novus Biologicals NB100-690) 1:200 in 3% BSA. Cells were washed well and incubated with goat anti-mouse 488 conjugated antibody (Thermo Fisher A-11001) for 4 h at room temperature. Cells were washed and incubated with CellMask™ Orange Actin Tracking Stain (Thermo Fisher A57244) at 1:1000 for 15 min, prior to further washing and imaging using the Operetta CLS system from Perkin Elmer (now Revvity).

#### Imaging analysis

The median of all cell data was determined per well. Any wells with < = 10 analysed field of view of less than 1000 cells per well were excluded. Initial scaling was undertaken to normalize the number of classified cells (proliferative, non-proliferative, apoptotic or enlarged) per well by replicate. This entailed calculating a per well scaling factor by dividing the number of classified objects per well by the total number of classified objects in all wells for the replicate (i.e. relative contribution). The number of objects classified as apoptotic, non-proliferative, proliferative, or enlarged was then divided by this factor. Any well containing less than 5% proliferating cells was excluded. To allow additional scaling by features, PCA plots were used to identify outlier control wells for removal, resulting in the exclusion of 6 control wells. In total, data from 4 independent replicates were taken forward for analysis.

#### Off-target score calculation

We calculated on-target scores for single guides using CRISOT [60] with default arguments. To generate off-target scores, we first identified putative off-target sites using cas-offinder [61]. For each identified site, we calculated both MIT [62] and CFD [63] off-target scores using the crisprScore package [64] in R (v4.3.2) (Additional File 1: Table S1). The resulting site-wise scores from both methods correlated strongly (Pearson R = 0.96). Both on- and off-target scores were aggregated by taking the maximum score over all putative off-target sites, prioritizing sites with zero mismatches. The entire workflow was reproducibly implemented using snakemake. Guide sequences were aligned to the human reference genome assembly GRCh38.

#### Comparison of screen hits to gene expression data

For the analysis in Fig. 7 we compared the 117 screen hits described above to expression data. More specifically, we identified genes that were ubiquitously expressed in normal tissues with a TPM > 1 for both members of the pair in GTEx expression data. We also identified genes where in tumours there was loss of expression in tumours in the pan-cancer analysis of whole cancer genomes (PCAWG) dataset where there was loss of expression of one member of the pair but not the other. The data for normal tissues came from GTEx release E-MTAB-5200 and the tumour data from 1350 patients from PCAWG https://dcc.icgc.org/pcawg was re-analysed through the Expression Atlases standardised pipelines and normalised across samples by patient/individual and had the accession E-MTAB-5423.

To identify tissue specific genetic interactions, we used a Type II ANOVA analysis. The GI effect score for each pair was treated as a dependent variable in a separate one-way ANOVA tests, with cancer type as an independent categorical variable. We used the Benjamini–Hochberg approach [65] to correct for multiple hypothesis testing, with results considered significant at a false discovery rate of < 10%. To assess the relationship between mRNA abundance and either GI scores or individual gene effect scores, we used Pearson correlation. For each of the gene pairs identified as synthetic lethal in at least three cell lines, we calculated the Pearson correlation coefficients between the mRNA expression levels of both genes in the pair and their corresponding GI scores. Additionally, we computed the correlation between the mRNA expression level of either gene and the gene effect score of its partner, in both directions. The significance of these correlations was determined by applying the Benjamini–Hochberg correction, with an FDR threshold of 10%. Data for this analysis was obtained from the DepMap portal (https://depmap.org/portal/), using the version 24Q2 Public dataset [66]. This included mRNA expression data (DepMap 24Q2 Public, OmicsExpressionProteinCodingGenesTPMLogp1) and gene effect scores (DepMap 24Q2 Public, CRISPRGeneEffect) for 26 cancer cell lines.

## Supplementary Information

Additional file1

Additional file2

Additional file3

## Data Availability

**All of the code in the manuscript is available for download from Github** [67] **and is also available via the Zenodo repository** [49] **. Codes is released under an MIT license.** —Jupyter Notebooks are available for the combinatorial CRISPR screen analysis: [https://github.com/team113sanger/harle_dgCRISPR_paralogs/blob/develop/combinatorial_crispr_screen_analysis/JupyterNotebooks](https:/github.com/team113sanger/harle_dgCRISPR_paralogs/blob/develop/combinatorial_crispr_screen_analysis/JupyterNotebooks). These documents allow analysis directly from the raw data downloaded from the ENA (01_Data_Preparation.ipynb), through QC and downstream analyses and post-processing.—Scripts used in the Notebooks can be found here: [https://github.com/team113sanger/harle_dgCRISPR_paralogs/tree/develop/combinatorial_crispr_screen_analysis/SCRIPTS](https:/github.com/team113sanger/harle_dgCRISPR_paralogs/tree/develop/combinatorial_crispr_screen_analysis/SCRIPTS)—Data not in ENA (e.g. metadata) but too large to store in the repository can be found on Figshare[68]: [https://figshare.com/articles/dataset/A_compendium_of_synthetic_lethal_gene_pairs_defined_by_extensive_combinatorial_pan-cancer_CRISPR_screening/25954027](https:/figshare.com/articles/dataset/A_compendium_of_synthetic_lethal_gene_pairs_defined_by_extensive_combinatorial_pan-cancer_CRISPR_screening/25954027)—Scripts for processing imaging data are here: https://github.com/team113sanger/harle_dgCRISPR_paralogs/tree/develop/imaging_analysis The sequencing data generated from the CRISPR screens in this study are available from the European Nucleotide Archive under accession: PRJEB60853[69]. RNA transcriptome data (except C092) were downloaded from the Broad DepMap portal[66]. RNA-Seq data for C092 is available from the European Genome-Phenome Archive (EGA) under accession: EGAS00001000815[70] and is managed by the Sanger Institute Data Access Committee (datasharing@sanger.ac.uk). All other data is available in the Supplementary information. Data from the TCGA[25,46], Zena Browser[45], GTEX[38] and SLKB[34] databases were obtained from those repositories. Jupyter Notebooks are available for the combinatorial CRISPR screen analysis:https://github.com/team113sanger/harle_dgCRISPR_paralogs/blob/develop/combinatorial_crispr_screen_analysis/JupyterNotebooks. These documents allow analysis directly from the raw data downloaded from the ENA (01_Data_Preparation.ipynb), through QC and downstream analyses and post-processing. Scripts used in the Notebooks can be found here:https://github.com/team113sanger/harle_dgCRISPR_paralogs/tree/develop/combinatorial_crispr_screen_analysis/SCRIPTS Data not in ENA (e.g. metadata) but too large to store in the repository can be found on Figshare [68]: https://figshare.com/articles/dataset/A_compendium_of_synthetic_lethal_gene_pairs_defined_by_extensive_combinatorial_pan-cancer_CRISPR_screening/25954027 Scripts for processing imaging data are here: https://github.com/team113sanger/harle_dgCRISPR_paralogs/tree/develop/imaging_analysis The sequencing data generated from the CRISPR screens in this study are available from the European Nucleotide Archive under accession: PRJEB60853 [69]. RNA transcriptome data (except C092) were downloaded from the Broad DepMap portal [66]. RNA-Seq data for C092 is available from the European Genome-Phenome Archive (EGA) under accession: EGAS00001000815 [70] and is managed by the Sanger Institute Data Access Committee (datasharing@sanger.ac.uk). All other data is available in the Supplementary information. Data from the TCGA [25, 46], Zena Browser [45], GTEX [38] and SLKB [34] databases were obtained from those repositories.
