# Supplementary material for: A compendium of synthetic lethal gene pairs defined by extensive combinatorial pan-cancer CRISPR screening
Source: Genome Biol. 2025 Sep 18;26:284. doi: 10.1186/s13059-025-03737-w (PMC12445041; doi:10.1186/s13059-025-03737-w)
Supplement: Supplementary file 2 — Additional file2 [file 13059_2025_3737_MOESM2_ESM.pdf]

A compendium of synthetic lethal gene pairs defined by extensive combinatorial pan-cancer CRISPR screening

Victoria Harle[1], Victoria Offord[1], Birkan Gökbağ[2], Lazaros Fotopoulos[3], Thomas Williams[3], Diana Alexander[1], Ishan Mehta[1], Nicola A. Thompson[1], Rebeca Olvera-León[1], Stefan Peidli[4], Vivek Iyer[1], Emanuel Gonçalves[1,5,6], Narod Kebabci[7,8], Barbara de Kegel[7,8], Joris van de Haar[9], Lang Li[2], Colm Ryan[7,8] & David J. Adams[1*]

[1]Wellcome Sanger Institute, Wellcome Trust Genome Campus, Hinxton, Cambridge, UK.
[2]Department of Biomedical Informatics. The Ohio State University, College of Medicine. Columbus, OH, OH 43210.
[3]Centre for Gene Therapy and Regenerative Medicine, King's College London 28th floor, Tower Wing, Guy's Hospital, Great Maze Pond, London.
[4]European Molecular Biology Laboratory (EMBL). Meyerhofstr. 1 69117. Heidelberg. Germany.
[5]Instituto Superior Técnico (IST), Universidade de Lisboa, 1049-001 Lisboa, Portugal.
[6]INESC-ID, 1000-029 Lisboa, Portugal.
[7]Conway Institute and School of Computer Science, University College Dublin, Dublin, Ireland; Systems Biology Ireland, University College Dublin, Dublin, Ireland.
[8]Conway Institute and School of Computer Science, University College Dublin, Dublin, Ireland; Science Foundation Ireland (SFI) Centre for Research Training in Genomics Data Science, University College Dublin, Dublin, Ireland.
[9]The Netherlands Cancer Institute. Plesmanlaan 121, 1066 CX Amsterdam, Netherlands.

Correspondence: Dr. David Adams: da1@sanger.ac.uk
Ph: +44 1223 496862

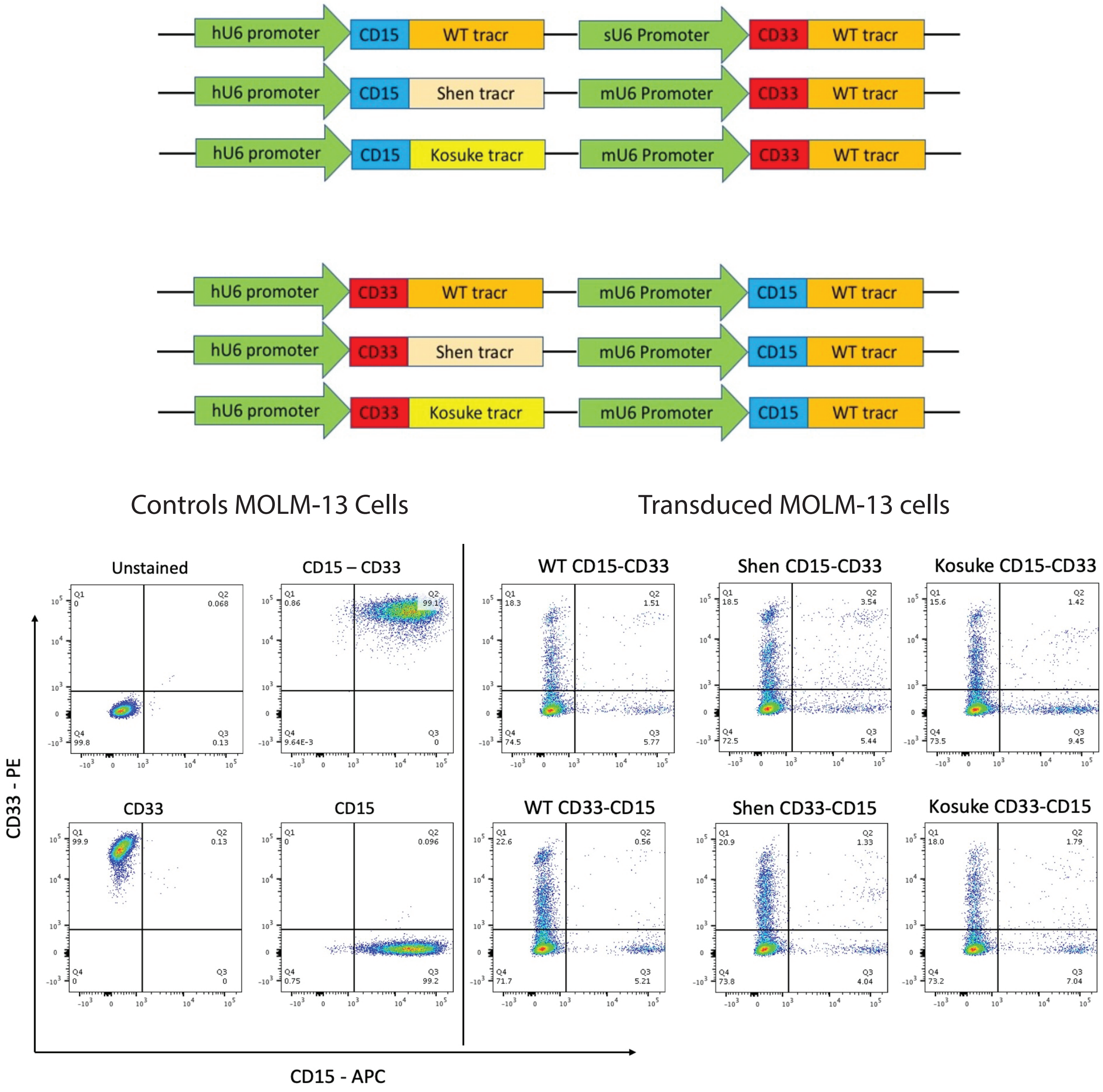

Additional File 2: Fig S1: **Validation of the dual gRNA CRISPR system.** Top: Vector designs showing the human (hU6) and mouse (mU6) U6 promoters and tracr sequences (Yusa and Shen)[1,2] arranged with the aim of reducing lentiviral recombination. Bottom: MOLM-13 cells express CD15 and CD33 on their cell surface. These cells were transduced with one of the vectors shown and analysed by flow cytometry. Controls and gating are shown on the left. There are some differences between the vectors used here and the one used for the final library (Additional File 3) but these experiments illustrate that the elements of the system work as expected.

Schematic of guide layout for cloning

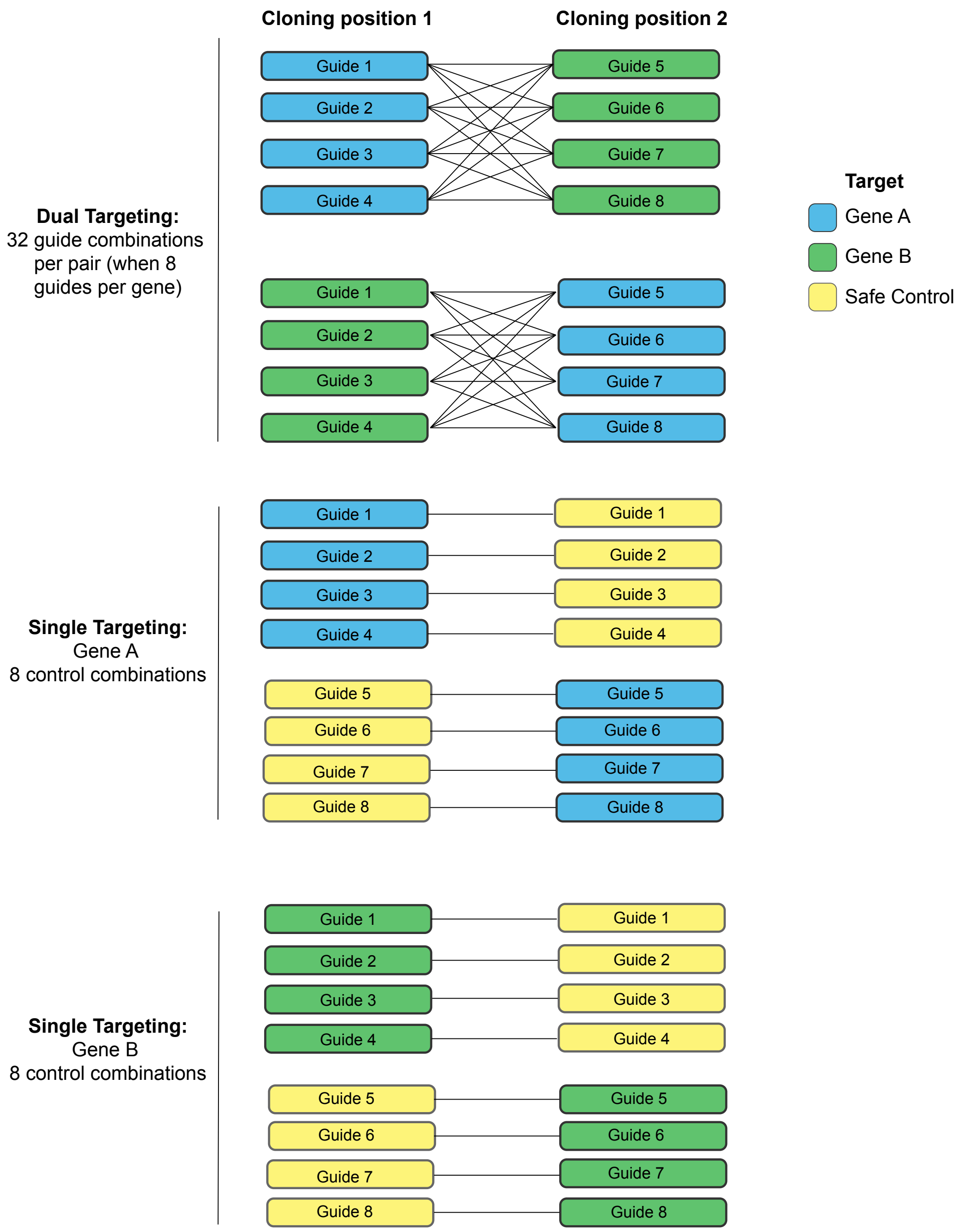

Additional File 2: Fig S2: Schematic of the guide layout/organisation in the library. The safe-targeting controls were collected from Morgens et al.,[3]. The guides used and their origin/source are provided in Additional File 1: Table S1.

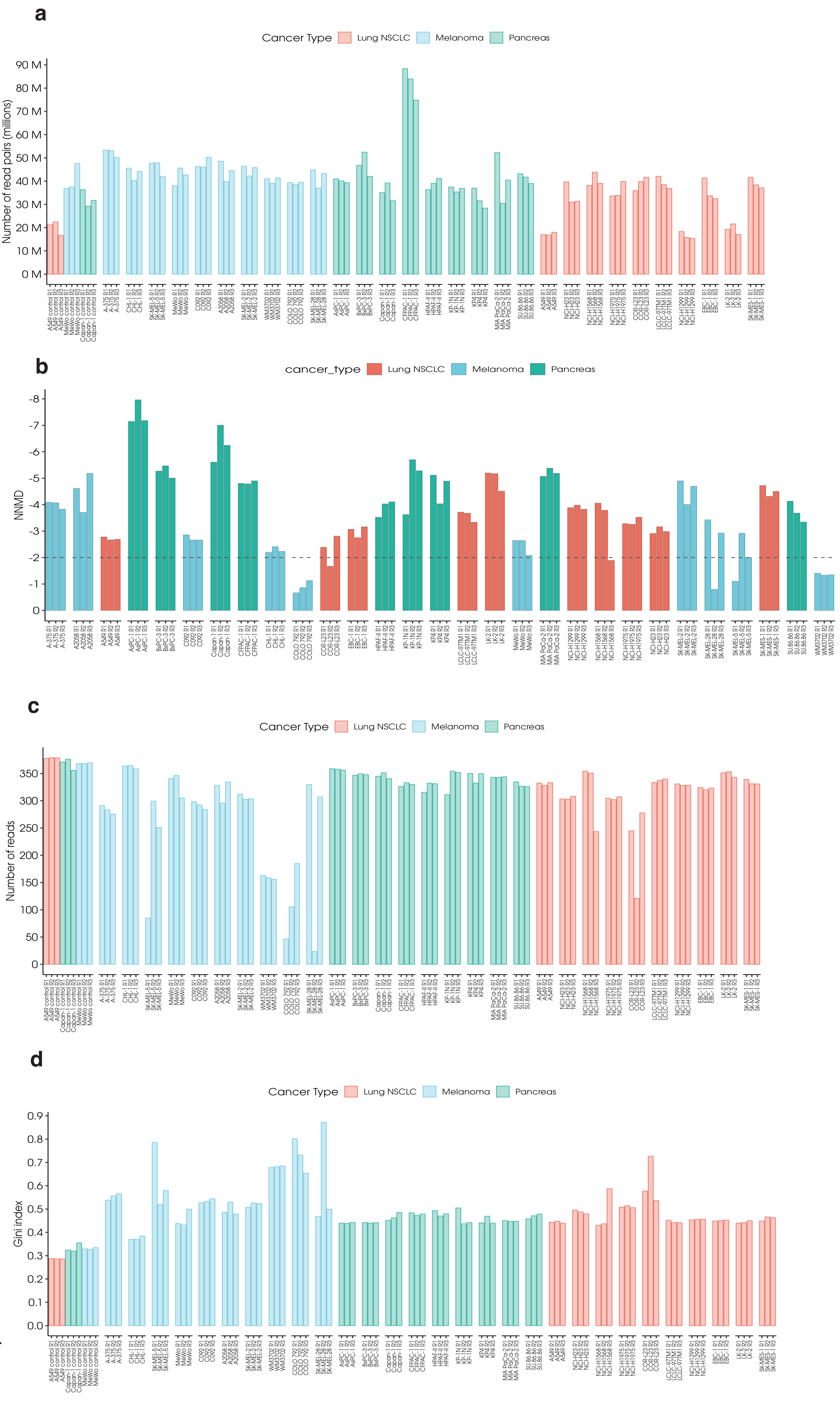
a
Cancer Type
Lung NSCLC
Melanoma
Pancreas
Number of read pairs (millions)
90 M
80 M
70 M
60 M
50 M
40 M
30 M
20 M
10 M
0 M
b
cancer_type
Lung NSCLC
Melanoma
Pancreas
NNMD
-8
-7
-6
-5
-4
-3
-2
-1
0
c
Cancer Type
Lung NSCLC
Melanoma
Pancreas
Number of reads
350
300
250
200
150
100
50
0
d
Cancer Type
Lung NSCLC
Melanoma
Pancreas
Gini index
0.9
0.8
0.7
0.6
0.5
0.4
0.3
0.2
0.1
0.0

Additional File 2: Fig S3: **Screen sequencing metrics**. A. This graph shows each replicate for each cell line and the number of read pairs generated. B. Null-normalised mean difference (NNMD) of the normalised fold changes with a cutoff of -2 coloured by cancer type. C. Median read count (read pairs) per gRNA pair (calculated from normalised counts). D. This graph shows the Gini index for each replicate. The colour indicates the origin of the cell line from lung (red), melanoma (blue) and pancreas (green).

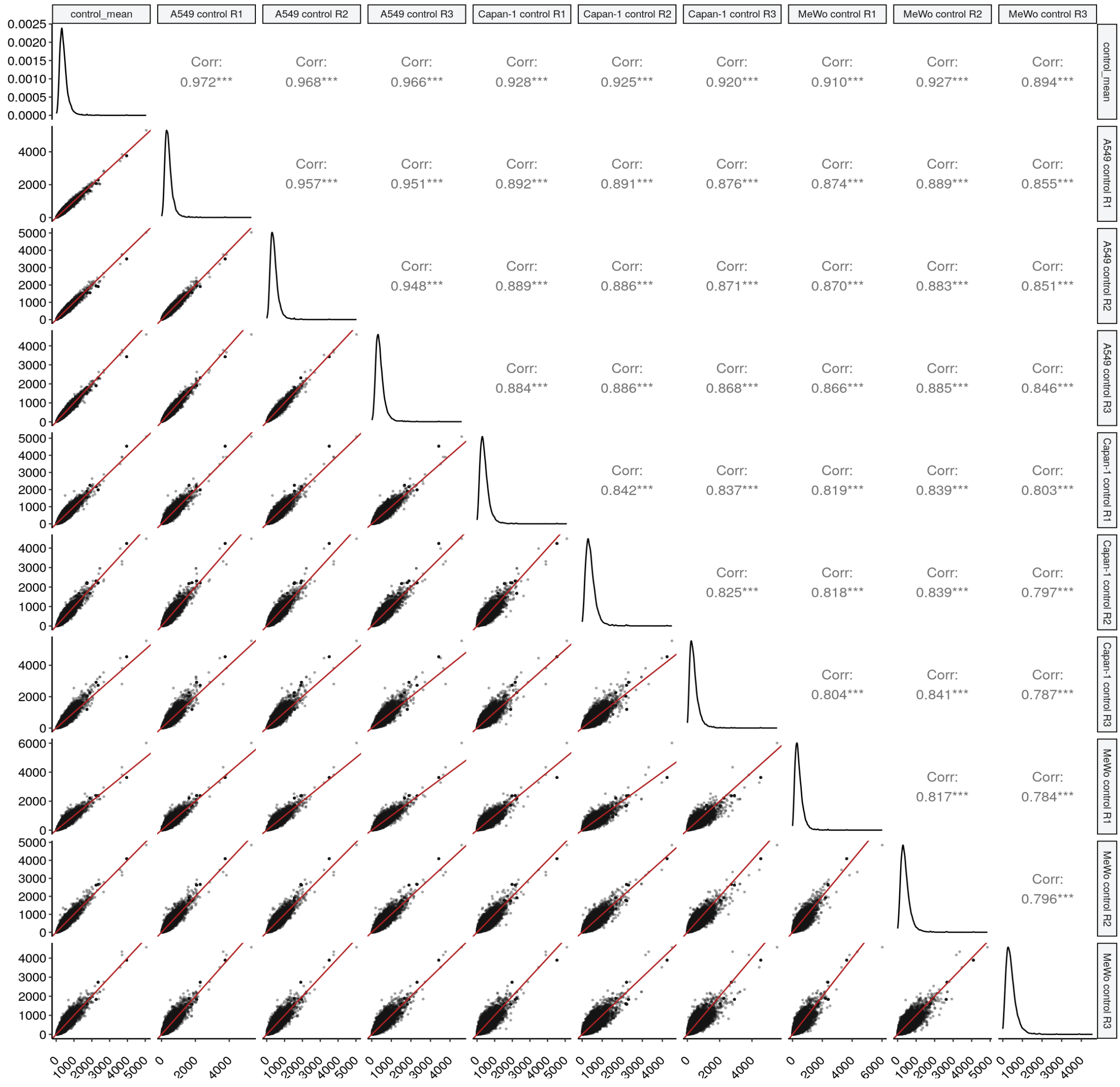

Additional file 2: Fig S4: **Control Replicate Analysis.** Spearman's correlation of the normalised counts between replicates from the three Cas9 wildtype lines (Capan1, A-549 and MeWo) and the averaged control (control_mean).

Broad DepMap

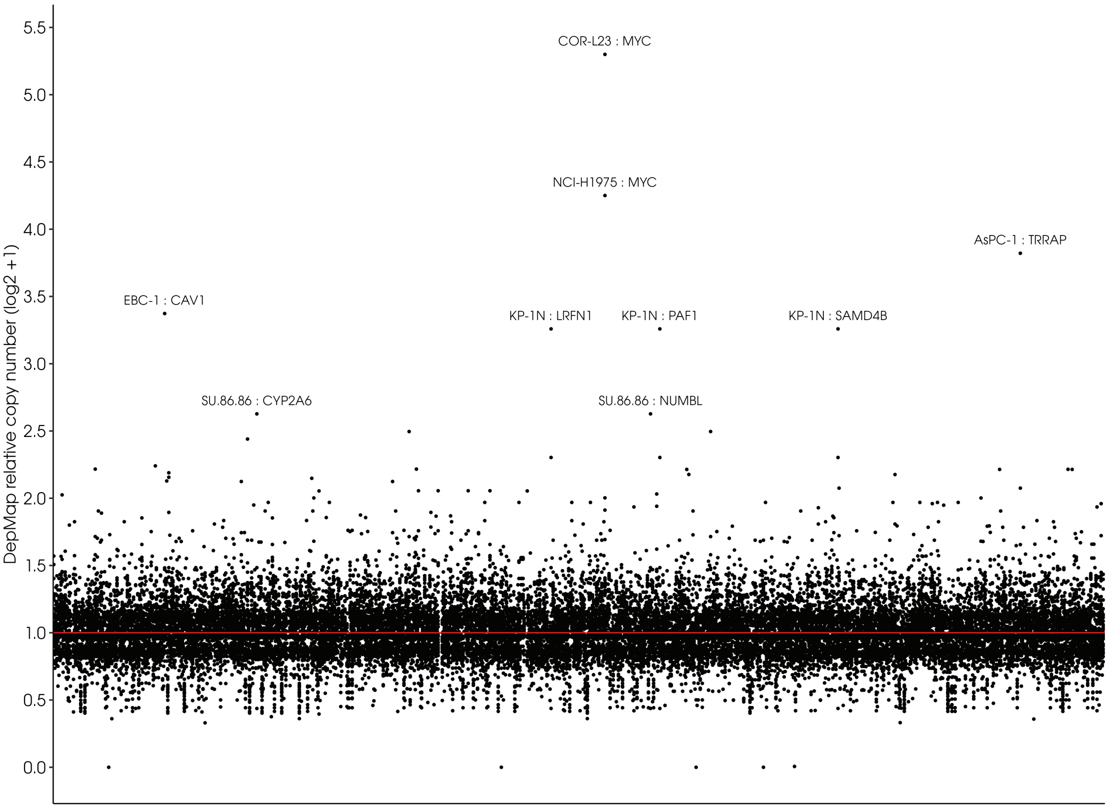

Cell Models Passport

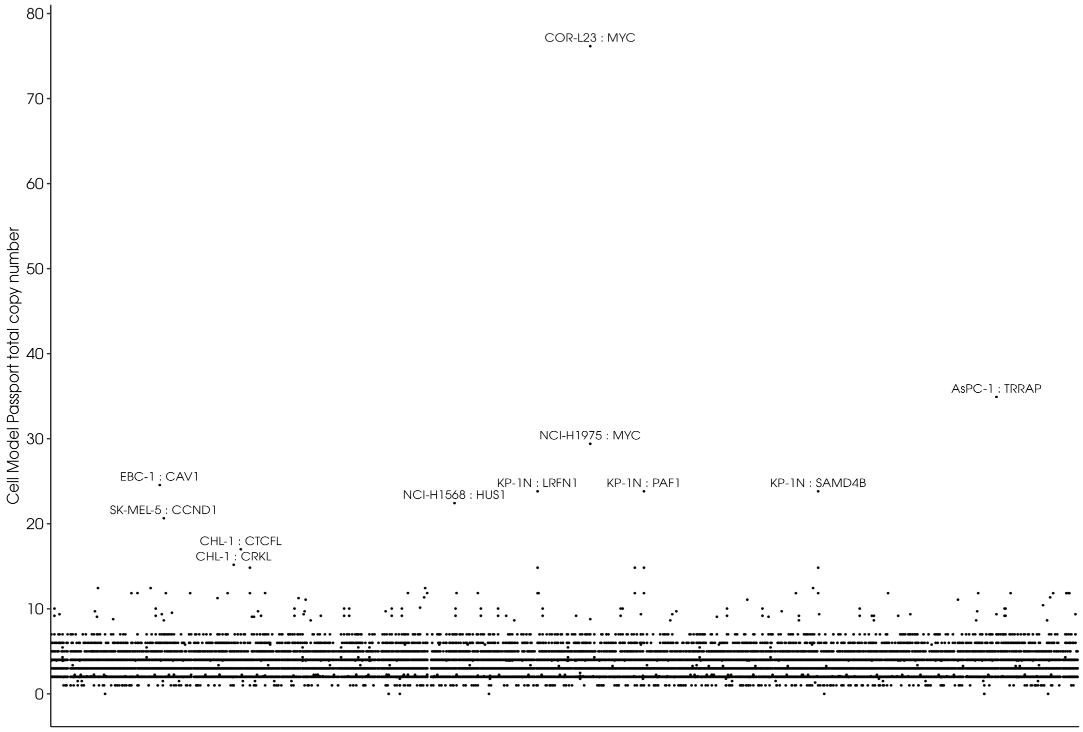

Genomic position

Additional File 2: Fig S5: **Genes in our combinatorial CRISPR library are generally not amplified in the cell lines we screened.** Shown is the pooled copy number for the cell lines screened with genes targeted in the library and the cell lines in which they show a copy number alteration shown. The data comes from DepMap and the Cell Models Passport as indicated.

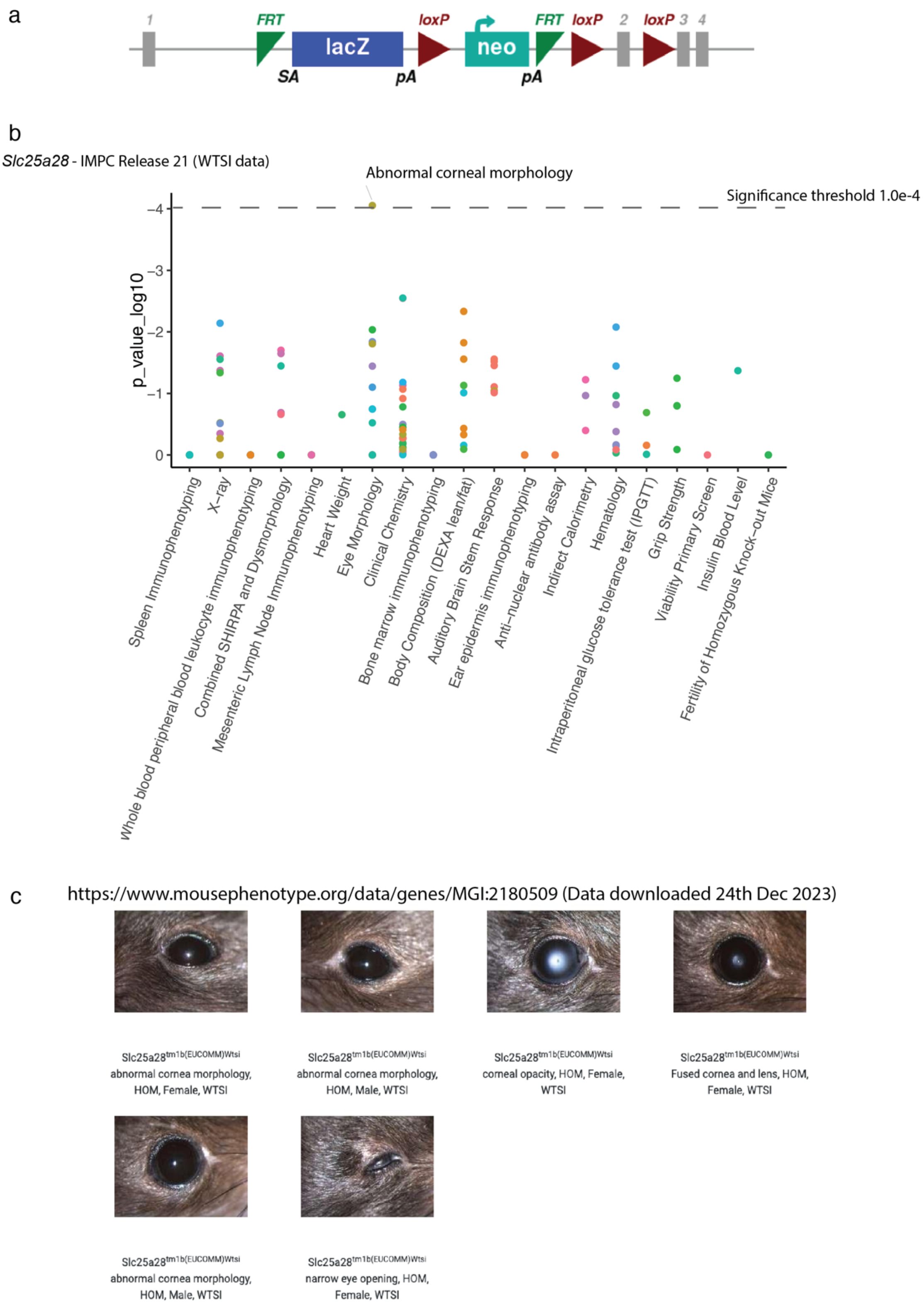

Additional file 2: Fig S6: **Phenotyping of the *Slc25a28* knockout line**. A. The design of the *Slc25a28* allele. Phenotyping was performed on the *tm1b* allele after Cre-mediated excision of the critical exon flanked by LoxP sites (exon 2)[4]. B. *Slc25a28* knockout animals were extensively phenotyped at the Wellcome Sanger Institute[5].

Shown are phenotypes tested and their P values. The data is from release 21 of the International Mouse Phenotying Consortium (IMPC)[6] with P values calculated using Phenstat (mixed modelling)[7] or a Chi-squared test for categorical data. Note: the only significant phenotype is an eye defect, as shown. Each of the dots represents a different phenotyping test performed in the phenotypic area shown on the X-axis. C. Slit lamp analysis of *Slc25a28* knockout mouse mutants showing a range of presentations including fused cornea, narrowed eye and corneal opacity. Eye phenotyping was performed as described previously[5].

REFERENCES

1. Shen JP, Zhao D, Sasik R, Luebeck J, Birmingham A, Bojorquez-Gomez A, et al. Combinatorial CRISPR–Cas9 screens for de novo mapping of genetic interactions. Nat Methods. 2017;14:573–6.

2. Koike-Yusa H, Li Y, Tan E-P, Velasco-Herrera MDC, Yusa K. Genome-wide recessive genetic screening in mammalian cells with a lentiviral CRISPR-guide RNA library. Nat Biotechnol. 2013;32:267–73.

3. Morgens DW, Wainberg M, Boyle EA, Ursu O, Araya CL, Tsui CK, et al. Genome-scale measurement of off-target activity using Cas9 toxicity in high-throughput screens. Nat Commun. 2017;8:15178.

4. Skarnes WC, Rosen B, West AP, Koutsourakis M, Bushell W, Iyer V, et al. A conditional knockout resource for the genome-wide study of mouse gene function. Nature. 2011;474:337–42.

5. White JK, Gerdin A-K, Karp NA, Ryder E, Buljan M, Bussell JN, et al. Genome-wide Generation and Systematic Phenotyping of Knockout Mice Reveals New Roles for Many Genes. Cell. 2013;154:452–64.

6. Consortium TIMP, Meehan TF, Conte N, West DB, Jacobsen JO, Mason J, et al. Disease model discovery from 3,328 gene knockouts by The International Mouse Phenotyping Consortium. Nat Genet. 2017;49:1231–8.

7. Kurbatova N, Mason JC, Morgan H, Meehan TF, Karp NA. PhenStat: A Tool Kit for Standardized Analysis of High Throughput Phenotypic Data. PLoS ONE. 2015;10:e0131274.
