# Supplementary material for: A compendium of synthetic lethal gene pairs defined by extensive combinatorial pan-cancer CRISPR screening
Source: Genome Biol. 2025 Sep 18;26:284. doi: 10.1186/s13059-025-03737-w (PMC12445041; doi:10.1186/s13059-025-03737-w)
Supplement: Supplementary file 3 — Additional file3 [file 13059_2025_3737_MOESM3_ESM.docx]

LOCUS Exported 8841 bp DNA circular SYN 02-APR-2020

DEFINITION synthetic circular DNA

ACCESSION .

VERSION .

KEYWORDS Paired Library Vector v2

SOURCE synthetic DNA construct

ORGANISM synthetic DNA construct

REFERENCE 1 (bases 1 to 8841)

AUTHORS .

TITLE Direct Submission

JOURNAL Exported Dec 23, 2023 from SnapGene Viewer 6.0.2

https://www.snapgene.com

FEATURES Location/Qualifiers

source 1..8841

/mol_type="other DNA"

/organism="synthetic DNA construct"

promoter 162..402

/label=hU6 promoter

misc_feature 412..431

/label=gRNA 1

misc_feature 432..523

/label=Modified gRNA scaffold

promoter 609..928

/label=mU6 promoter

misc_feature 930..949

/label=gRNA 2

misc_feature 950..1025

/label=gRNA scaffold

misc_feature 1081..1198

/label=cPPT/CTS

/note="central polypurine tract and central termination

sequence of HIV-1"

promoter 1337..2515

/label=EF-1-alpha promoter

/note="strong constitutive promoter for human elongation

factor EF-1-alpha"

intron 1568..2506

/label=EF-1-alpha intron A

/note="intron upstream of the start codon of human

EF-1-alpha"

CDS 2528..3127

/codon_start=1

/gene="pac from Streptomyces alboniger"

/product="puromycin N-acetyltransferase"

/label=PuroR

/note="confers resistance to puromycin"

/translation="MTEYKPTVRLATRDDVPRAVRTLAAAFADYPATRHTVDPDRHIER

VTELQELFLTRVGLDIGKVWVADDGAAVAVWTTPESVEAGAVFAEIGPRMAELSGSRLA

AQQQMEGLLAPHRPKEPAWFLATVGVSPDHQGKGLGSAVVLPGVEAAERAGVPAFLETS

APRNLPFYERLGFTVTADVEVPEGPRTWCMTRKPGA"

misc_feature 3143..3731

/label=WPRE

/note="woodchuck hepatitis virus posttranscriptional

regulatory element"

CDS complement(3614..3625)

/codon_start=1

/product="Factor Xa recognition and cleavage site"

/label=Factor Xa site

/translation="IEGR"

LTR 3803..4036

/label=3' LTR (Delta-U3)

/note="self-inactivating 3' long terminal repeat (LTR) from

HIV-1"

polyA_signal 4114..4235

/label=SV40 poly(A) signal

/note="SV40 polyadenylation signal"

rep_origin 4275..4410

/label=SV40 ori

/note="SV40 origin of replication"

promoter complement(4431..4449)

/label=T7 promoter

/note="promoter for bacteriophage T7 RNA polymerase"

primer_bind complement(4459..4475)

/label=M13 fwd

/note="common sequencing primer, one of multiple similar

variants"

rep_origin 4617..5072

/direction=RIGHT

/label=f1 ori

/note="f1 bacteriophage origin of replication; arrow

indicates direction of (+) strand synthesis"

promoter 5098..5202

/gene="bla"

/label=AmpR promoter

CDS 5203..6063

/codon_start=1

/gene="bla"

/product="beta-lactamase"

/label=AmpR

/note="confers resistance to ampicillin, carbenicillin, and

related antibiotics"

/translation="MSIQHFRVALIPFFAAFCLPVFAHPETLVKVKDAEDQLGARVGYI

ELDLNSGKILESFRPEERFPMMSTFKVLLCGAVLSRIDAGQEQLGRRIHYSQNDLVEYS

PVTEKHLTDGMTVRELCSAAITMSDNTAANLLLTTIGGPKELTAFLHNMGDHVTRLDRW

EPELNEAIPNDERDTTMPVAMATTLRKLLTGELLTLASRQQLIDWMEADKVAGPLLRSA

LPAGWFIADKSGAGERGSRGIIAALGPDGKPSRIVVIYTTGSQATMDERNRQIAEIGAS

LIKHW"

rep_origin 6234..6822

/direction=RIGHT

/label=ori

/note="high-copy-number ColE1/pMB1/pBR322/pUC origin of

replication"

protein_bind 7110..7131

/label=CAP binding site

/bound_moiety="E. coli catabolite activator protein"

/note="CAP binding activates transcription in the presence

of cAMP."

promoter 7146..7176

/label=lac promoter

/note="promoter for the E. coli lac operon"

protein_bind 7184..7200

/label=lac operator

/bound_moiety="lac repressor encoded by lacI"

/note="The lac repressor binds to the lac operator to

inhibit transcription in E. coli. This inhibition can be

relieved by adding lactose or

isopropyl-beta-D-thiogalactopyranoside (IPTG)."

primer_bind 7208..7224

/label=M13 rev

/note="common sequencing primer, one of multiple similar

variants"

promoter 7245..7263

/label=T3 promoter

/note="promoter for bacteriophage T3 RNA polymerase"

promoter 7291..7517

/label=RSV promoter

/note="Rous sarcoma virus enhancer/promoter"

LTR 7518..7698

/label=5' LTR (truncated)

/note="truncated 5' long terminal repeat (LTR) from HIV-1"

misc_feature 7745..7870

/label=HIV-1 Psi

/note="packaging signal of human immunodeficiency virus

type 1"

misc_feature 8363..8596

/label=RRE

/note="The Rev response element (RRE) of HIV-1 allows for

Rev-dependent mRNA export from the nucleus to the

cytoplasm."

CDS 8781..8825

/codon_start=1

/product="antigenic peptide corresponding to amino acids

655 to 669 of the HIV envelope protein gp41 (Lutje Hulsik

et al., 2013)"

/label=gp41 peptide

/note="recognized by the 2H10 single-chain llama nanobody"

/translation="KNEQELLELDKWASL"

ORIGIN

1 taacaaattg gctgtggtat ataaaattat tcataatgat agtaggaggc ttggtaggtt

61 taagaatagt ttttgctgta ctttctatag tgaatagagt taggcaggga tattcaccat

121 tatcgtttca gacccacctc ccaaccccga ggggacccag agagggccta tttcccatga

181 ttccttcata tttgcatata cgatacaagg ctgttagaga gataattaga attaatttga

241 ctgtaaacac aaagatatta gtacaaaata cgtgacgtag aaagtaataa tttcttgggt

301 agtttgcagt tttaaaatta tgttttaaaa tggactatca tatgcttacc gtaacttgaa

361 agtatttcga tttcttggct ttatatatct tgtggaaagg acgaaacacc gtggggtgat

421 tatgagcacc ggtttcagag ctatgctgga aactgcatag caagttgaaa taaggctagt

481 ccgttatcaa cttgaaaaag tggcaccgag tcggtgcttt tttgaactag ctgtagatct

541 aggaagttaa aattgtcgta ggagcttttt agcgcgtgcg ccaattctgc agacaaatgg

601 ctctagatct agagatccga cgccgccatc tctaggcccg cgccggcccc ctccacagac

661 ttgtgggaga agctcggcta ctcccctgcc ccggttaatt tgcatataat atttcctagt

721 aactatagag gcttaatgtg cgataaaaga cagataatct gttcttttta atactagcta

781 cattttacat gataggcttg gatttctata agagatacaa atactaaatt attattttaa

841 aaaacagcac aaaaggaaac tcaccctaac tgtaaagtaa ttgtgtgttt tgagactata

901 aatatgcatg cgagaaaagc cttgtttggc gcgtcctacg gagaggctcg ttttagagct

961 agaaatagca agttaaaata aggctagtcc gttatcaact tgaaaaagtg gcaccgagtc

1021 ggtgcttttt taagcttggc gtaactagat cttgagacaa atggcagtat tcatccacaa

1081 ttttaaaaga aaagggggga ttggggggta cagtgcaggg gaaagaatag tagacataat

1141 agcaacagac atacaaacta aagaattaca aaaacaaatt acaaaaattc aaaattttcg

1201 ggtttattac agggacagca gagatccact ttggcgccgg ctcgaggggg cccgggtgca

1261 aagatggata aagttttaaa cagagaggaa tctttgcagc taatggacct tctaggtctt

1321 gaaaggagtg ggaattggct ccggtgcccg tcagtgggca gagcgcacat cgcccacagt

1381 ccccgagaag ttggggggag gggtcggcaa ttgatccggt gcctagagaa ggtggcgcgg

1441 ggtaaactgg gaaagtgatg tcgtgtactg gctccgcctt tttcccgagg gtgggggaga

1501 accgtatata agtgcagtag tcgccgtgaa cgttcttttt cgcaacgggt ttgccgccag

1561 aacacaggta agtgccgtgt gtggttcccg cgggcctggc ctctttacgg gttatggccc

1621 ttgcgtgcct tgaattactt ccacctggct gcagtacgtg attcttgatc ccgagcttcg

1681 ggttggaagt gggtgggaga gttcgaggcc ttgcgcttaa ggagcccctt cgcctcgtgc

1741 ttgagttgag gcctggcctg ggcgctgggg ccgccgcgtg cgaatctggt ggcaccttcg

1801 cgcctgtctc gctgctttcg ataagtctct agccatttaa aatttttgat gacctgctgc

1861 gacgcttttt ttctggcaag atagtcttgt aaatgcgggc caagatctgc acactggtat

1921 ttcggttttt ggggccgcgg gcggcgacgg ggcccgtgcg tcccagcgca catgttcggc

1981 gaggcggggc ctgcgagcgc ggccaccgag aatcggacgg gggtagtctc aagctggccg

2041 gcctgctctg gtgcctggcc tcgcgccgcc gtgtatcgcc ccgccctggg cggcaaggct

2101 ggcccggtcg gcaccagttg cgtgagcgga aagatggccg cttcccggcc ctgctgcagg

2161 gagctcaaaa tggaggacgc ggcgctcggg agagcgggcg ggtgagtcac ccacacaaag

2221 gaaaagggcc tttccgtcct cagccgtcgc ttcatgtgac tccacggagt accgggcgcc

2281 gtccaggcac ctcgattagt tctcgagctt ttggagtacg tcgtctttag gttgggggga

2341 ggggttttat gcgatggagt ttccccacac tgagtgggtg gagactgaag ttaggccagc

2401 ttggcacttg atgtaattct ccttggaatt tgcccttttt gagtttggat cttggttcat

2461 tctcaagcct cagacagtgg ttcaaagttt ttttcttcca tttcaggtgt cgtgacgtac

2521 ggccaccatg accgagtaca agcccacggt gcgcctcgcc acccgcgacg acgtccccag

2581 ggccgtacgc accctcgccg ccgcgttcgc cgactacccc gccacgcgcc acaccgtcga

2641 tccggaccgc cacatcgagc gggtcaccga gctgcaagaa ctcttcctca cgcgcgtcgg

2701 gctcgacatc ggcaaggtgt gggtcgcgga cgacggcgcc gccgtggcgg tctggaccac

2761 gccggagagc gtcgaagcgg gggcggtgtt cgccgagatc ggcccgcgca tggccgagtt

2821 gagcggttcc cggctggccg cgcagcaaca gatggaaggc ctcctggcgc cgcaccggcc

2881 caaggagccc gcgtggttcc tggccaccgt cggagtctcg cccgaccacc agggcaaggg

2941 tctgggcagc gccgtcgtgc tccccggagt ggaggcggcc gagcgcgccg gggtgcccgc

3001 cttcctggag acctccgcgc cccgcaacct ccccttctac gagcggctcg gcttcaccgt

3061 caccgccgac gtcgaggtgc ccgaaggacc gcgcacctgg tgcatgaccc gcaagcccgg

3121 tgcctgaacg cgttaagtcg acaatcaacc tctggattac aaaatttgtg aaagattgac

3181 tggtattctt aactatgttg ctccttttac gctatgtgga tacgctgctt taatgccttt

3241 gtatcatgct attgcttccc gtatggcttt cattttctcc tccttgtata aatcctggtt

3301 gctgtctctt tatgaggagt tgtggcccgt tgtcaggcaa cgtggcgtgg tgtgcactgt

3361 gtttgctgac gcaaccccca ctggttgggg cattgccacc acctgtcagc tcctttccgg

3421 gactttcgct ttccccctcc ctattgccac ggcggaactc atcgccgcct gccttgcccg

3481 ctgctggaca ggggctcggc tgttgggcac tgacaattcc gtggtgttgt cggggaaatc

3541 atcgtccttt ccttggctgc tcgcctgtgt tgccacctgg attctgcgcg ggacgtcctt

3601 ctgctacgtc ccttcggccc tcaatccagc ggaccttcct tcccgcggcc tgctgccggc

3661 tctgcggcct cttccgcgtc ttcgccttcg ccctcagacg agtcggatct ccctttgggc

3721 cgcctccccg cgtcgacttt aagaccaatg acttacaagg cagctgtaga tcttagccac

3781 tttttaaaag aaaagggggg actggaaggg ctaattcact cccaacgaag acaagatctg

3841 ctttttgctt gtactgggtc tctctggtta gaccagatct gagcctggga gctctctggc

3901 taactaggga acccactgct taagcctcaa taaagcttgc cttgagtgct tcaagtagtg

3961 tgtgcccgtc tgttgtgtga ctctggtaac tagagatccc tcagaccctt ttagtcagtg

4021 tggaaaatct ctagcagtac gtatagtagt tcatgtcatc ttattattca gtatttataa

4081 cttgcaaaga aatgaatatc agagagtgag aggaacttgt ttattgcagc ttataatggt

4141 tacaaataaa gcaatagcat cacaaatttc acaaataaag catttttttc actgcattct

4201 agttgtggtt tgtccaaact catcaatgta tcttatcatg tctggctcta gctatcccgc

4261 ccctaactcc gcccatcccg cccctaactc cgcccagttc cgcccattct ccgccccatg

4321 gctgactaat tttttttatt tatgcagagg ccgaggccgc ctcggcctct gagctattcc

4381 agaagtagtg aggaggcttt tttggaggcc tagggacgta cccaattcgc cctatagtga

4441 gtcgtattac gcgcgctcac tggccgtcgt tttacaacgt cgtgactggg aaaaccctgg

4501 cgttacccaa cttaatcgcc ttgcagcaca tccccctttc gccagctggc gtaatagcga

4561 agaggcccgc accgatcgcc cttcccaaca gttgcgcagc ctgaatggcg aatgggacgc

4621 gccctgtagc ggcgcattaa gcgcggcggg tgtggtggtt acgcgcagcg tgaccgctac

4681 acttgccagc gccctagcgc ccgctccttt cgctttcttc ccttcctttc tcgccacgtt

4741 cgccggcttt ccccgtcaag ctctaaatcg ggggctccct ttagggttcc gatttagtgc

4801 tttacggcac ctcgacccca aaaaacttga ttagggtgat ggttcacgta gtgggccatc

4861 gccctgatag acggtttttc gccctttgac gttggagtcc acgttcttta atagtggact

4921 cttgttccaa actggaacaa cactcaaccc tatctcggtc tattcttttg atttataagg

4981 gattttgccg atttcggcct attggttaaa aaatgagctg atttaacaaa aatttaacgc

5041 gaattttaac aaaatattaa cgcttacaat ttaggtggca cttttcgggg aaatgtgcgc

5101 ggaaccccta tttgtttatt tttctaaata cattcaaata tgtatccgct catgagacaa

5161 taaccctgat aaatgcttca ataatattga aaaaggaaga gtatgagtat tcaacatttc

5221 cgtgtcgccc ttattccctt ttttgcggca ttttgccttc ctgtttttgc tcacccagaa

5281 acgctggtga aagtaaaaga tgctgaagat cagttgggtg cacgagtggg ttacatcgaa

5341 ctggatctca acagcggtaa gatccttgag agttttcgcc ccgaagaacg ttttccaatg

5401 atgagcactt ttaaagttct gctatgtggc gcggtattat cccgtattga cgccgggcaa

5461 gagcaactcg gtcgccgcat acactattct cagaatgact tggttgagta ctcaccagtc

5521 acagaaaagc atcttacgga tggcatgaca gtaagagaat tatgcagtgc tgccataacc

5581 atgagtgata acactgcggc caacttactt ctgacaacga tcggaggacc gaaggagcta

5641 accgcttttt tgcacaacat gggggatcat gtaactcgcc ttgatcgttg ggaaccggag

5701 ctgaatgaag ccataccaaa cgacgagcgt gacaccacga tgcctgtagc aatggcaaca

5761 acgttgcgca aactattaac tggcgaacta cttactctag cttcccggca acaattaata

5821 gactggatgg aggcggataa agttgcagga ccacttctgc gctcggccct tccggctggc

5881 tggtttattg ctgataaatc tggagccggt gagcgtgggt ctcgcggtat cattgcagca

5941 ctggggccag atggtaagcc ctcccgtatc gtagttatct acacgacggg gagtcaggca

6001 actatggatg aacgaaatag acagatcgct gagataggtg cctcactgat taagcattgg

6061 taactgtcag accaagttta ctcatatata ctttagattg atttaaaact tcatttttaa

6121 tttaaaagga tctaggtgaa gatccttttt gataatctca tgaccaaaat cccttaacgt

6181 gagttttcgt tccactgagc gtcagacccc gtagaaaaga tcaaaggatc ttcttgagat

6241 cctttttttc tgcgcgtaat ctgctgcttg caaacaaaaa aaccaccgct accagcggtg

6301 gtttgtttgc cggatcaaga gctaccaact ctttttccga aggtaactgg cttcagcaga

6361 gcgcagatac caaatactgt tcttctagtg tagccgtagt taggccacca cttcaagaac

6421 tctgtagcac cgcctacata cctcgctctg ctaatcctgt taccagtggc tgctgccagt

6481 ggcgataagt cgtgtcttac cgggttggac tcaagacgat agttaccgga taaggcgcag

6541 cggtcgggct gaacgggggg ttcgtgcaca cagcccagct tggagcgaac gacctacacc

6601 gaactgagat acctacagcg tgagctatga gaaagcgcca cgcttcccga agggagaaag

6661 gcggacaggt atccggtaag cggcagggtc ggaacaggag agcgcacgag ggagcttcca

6721 gggggaaacg cctggtatct ttatagtcct gtcgggtttc gccacctctg acttgagcgt

6781 cgatttttgt gatgctcgtc aggggggcgg agcctatgga aaaacgccag caacgcggcc

6841 tttttacggt tcctggcctt ttgctggcct tttgctcaca tgttctttcc tgcgttatcc

6901 cctgattctg tggataaccg tattaccgcc tttgagtgag ctgataccgc tcgccgcagc

6961 cgaacgaccg agcgcagcga gtcagtgagc gaggaagcgg aagagcgccc aatacgcaaa

7021 ccgcctctcc ccgcgcgttg gccgattcat taatgcagct ggcacgacag gtttcccgac

7081 tggaaagcgg gcagtgagcg caacgcaatt aatgtgagtt agctcactca ttaggcaccc

7141 caggctttac actttatgct tccggctcgt atgttgtgtg gaattgtgag cggataacaa

7201 tttcacacag gaaacagcta tgaccatgat tacgccaagc gcgcaattaa ccctcactaa

7261 agggaacaaa agctggagct gcaagcttaa tgtagtctta tgcaatactc ttgtagtctt

7321 gcaacatggt aacgatgagt tagcaacatg ccttacaagg agagaaaaag caccgtgcat

7381 gccgattggt ggaagtaagg tggtacgatc gtgccttatt aggaaggcaa cagacgggtc

7441 tgacatggat tggacgaacc actgaattgc cgcattgcag agatattgta tttaagtgcc

7501 tagctcgata cataaacggg tctctctggt tagaccagat ctgagcctgg gagctctctg

7561 gctaactagg gaacccactg cttaagcctc aataaagctt gccttgagtg cttcaagtag

7621 tgtgtgcccg tctgttgtgt gactctggta actagagatc cctcagaccc ttttagtcag

7681 tgtggaaaat ctctagcagt ggcgcccgaa cagggacttg aaagcgaaag ggaaaccaga

7741 ggagctctct cgacgcagga ctcggcttgc tgaagcgcgc acggcaagag gcgaggggcg

7801 gcgactggtg agtacgccaa aaattttgac tagcggaggc tagaaggaga gagatgggtg

7861 cgagagcgtc agtattaagc gggggagaat tagatcgcga tgggaaaaaa ttcggttaag

7921 gccaggggga aagaaaaaat ataaattaaa acatatagta tgggcaagca gggagctaga

7981 acgattcgca gttaatcctg gcctgttaga aacatcagaa ggctgtagac aaatactggg

8041 acagctacaa ccatcccttc agacaggatc agaagaactt agatcattat ataatacagt

8101 agcaaccctc tattgtgtgc atcaaaggat agagataaaa gacaccaagg aagctttaga

8161 caagatagag gaagagcaaa acaaaagtaa gaccaccgca cagcaagcgg ccgctgatct

8221 tcagacctgg aggaggagat atgagggaca attggagaag tgaattatat aaatataaag

8281 tagtaaaaat tgaaccatta ggagtagcac ccaccaaggc aaagagaaga gtggtgcaga

8341 gagaaaaaag agcagtggga ataggagctt tgttccttgg gttcttggga gcagcaggaa

8401 gcactatggg cgcagcgtca atgacgctga cggtacaggc cagacaatta ttgtctggta

8461 tagtgcagca gcagaacaat ttgctgaggg ctattgaggc gcaacagcat ctgttgcaac

8521 tcacagtctg gggcatcaag cagctccagg caagaatcct ggctgtggaa agatacctaa

8581 aggatcaaca gctcctgggg atttggggtt gctctggaaa actcatttgc accactgctg

8641 tgccttggaa tgctagttgg agtaataaat ctctggaaca gatttggaat cacacgacct

8701 ggatggagtg ggacagagaa attaacaatt acacaagctt aatacactcc ttaattgaag

8761 aatcgcaaaa ccagcaagaa aagaatgaac aagaattatt ggaattagat aaatgggcaa

8821 gtttgtggaa ttggtttaac a

//
